# Donor Acceptor Complexes between the Chalcogen Fluorides SF_2_, SeF_2_, SeF_4_ and TeF_4_ and an N‐Heterocyclic Carbene

**DOI:** 10.1002/chem.202201023

**Published:** 2022-06-20

**Authors:** Pascal Komorr, Marian Olaru, Emanuel Hupf, Stefan Mebs, Jens Beckmann

**Affiliations:** ^1^ Institut für Anorganische Chemie und Kristallographie Universität Bremen Leobener Straße 7 28359 Bremen Germany; ^2^ Institut für Experimentalphysik Freie Universität Berlin Arnimallee 14 14195 Berlin Germany

**Keywords:** N-heterocyclic carbine, fluorine, selenium, sulphur, tellurium

## Abstract

The majority of binary chalcogen fluorides are fiercely reactive and extremely difficult to handle. Here, we show that access to crystalline donor‐acceptor complexes between chalcogen difluorides (sulfur, selenium) and tetrafluorides (selenium, tellurium) with the N‐heterocyclic carbene (NHC) 1,3‐bis(2,6‐diisopropylphenyl)imidazol‐2‐ylidene (IPr) is possible conveniently and safely without the need to generate the highly unstable EF_2_ (E=S, Se) or the very toxic and corrosive SeF_4_.

## Graphical abstract

**Donor‐acceptor complexes** between the carbene 1,3‐bis(2,6‐diisopropylphenyl)imidazol‐2‐ylidene (IPr) and EF_2_ (E=S, Se) or EF_4_ (E=Se, Te) were conveniently synthesized. The compounds were structurally investigated by single crystal X‐ray diffraction and a suite of density functional theory (DFT) methods were employed in order to elucidate fine electronic details of the chemical environment around the central chalcogen atoms.

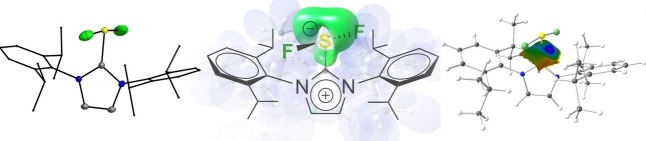

Amongst the many known chalcogen fluorides, only the tetrafluorides EF_4_ and hexafluorides EF_6_ (E=S, Se, Te) are readily accessible and structurally characterized. Electron diffraction studies of SF_4_,^[1]^ SeF_4_ ^[2]^ and TeF_4_ ^[3]^ reveal that they adopt pseudo trigonal bipyramidal structures with C_2v_ symmetry in the gas phase. These so‐called ‘seesaw’ structures are consistent with the valence shell electron pair repulsion (VESPR) model and the results of quantum chemical calculations.^[4]^ Owing to their inherent Lewis acidity, the same species are aggregated in condensed phase. The solid‐state structures of SF_4_,^[5]^ SeF_4_ ^[6]^ and TeF_4_[^6^, ^7^] established by single crystal X‐ray diffraction show an increasing degree of aggregation of the molecular entities by bridging fluorine atoms, which correlates well with increasing atomic number. In nonpolar solvents, the aggregation is retained in concentrated solutions, whereas monomers exist in high dilution. According to ^19^F NMR studies, the pseudo trigonal bipyramidal structures of these SF_4_,^[8]^ SeF_4_ ^[9]^ and TeF_4_ ^[10]^ monomers are fluxional with the axial and equatorial fluorine atoms being in rapid exchange at room temperature on the NMR scale, which has been attributed to Berry pseudorotation. At around −100 °C, the exchange can be slowed down for SF_4_ and SeF_4_, but not for TeF_4_. In polar solvents or the presence of donor molecules, Lewis pair complexes are formed, some of which have been isolated and fully characterized. For SF_4_, these include complexes with triethylamine,^[11]^ pyridine, 4‐methylpyrdine, DMAP,^[12]^ the mono‐protonated bipyridinium ion,^[13]^ THF, cyclobutanone, DME^[14]^ DABCO and urotropine.^[15]^ For TeF_4_, these include THF, toluene[^10^, ^16^] dioxane, DME^[16]^ and OPR_3_ (R=Me, Ph).^[17]^ In earlier work, it was concluded that TeF_4_ in certain donor solvents would undergo electrolytic dissociation into [TeF_3_(solvent)_n_]^+^ cations and the [TeF_5_]^–^ anion, however, this was never unambiguously proven.^[18]^ For SeF_4_, no donor acceptor complexes have been structurally characterised yet.[^19^, ^20^] In recent years, the bond situation in donor acceptor complexes of EF_4_ (E=S, Se, Te) with ammonia, amines and pyridines was investigated by quantum chemical calculations.[^21^, ^22^, ^23^] One of these studies also addressed the stability of complexes between EF_6_ (E=S, Se, Te) with ammonia, which was predicted to be very weak. The reaction of TeF_6_ with trimethylamine proceeded with reduction at tellurium and gave rise to the formation of [Me_2_NCH_2_NMe_3_][TeF_5_].^[24]^ The inherently unstable difluorides SF_2_ ^[25]^ and SeF_2_ ^[26]^ were generated photochemically and characterized in an argon matrix,^[27]^ whereas TeF_2_ is still unknown. For bulk SF_2_, an equilibrium was established with the mixed valent FSSF_3_, which, however, irreversibly converts into SSF_2_ and SF_4_.[^28^, ^29^] N‐heterocyclic carbenes (NHCs) are indispensable tools for the stabilization of low‐valent and Lewis acidic main group compounds.^[30]^ Surprisingly, there is only one claim of a donor acceptor complex between an NHC and a chalcogen fluoride, namely, (I^*i*^Pr_2_Me_2_)SF_2_, which, however, was not structurally characterized (see below).^[31]^ The lack of such donor acceptor complexes is even more surprising as related complexes involving higher chalcogen halides, such as (NHC)EX_2_ (E=S, Se, Te; X=Cl, Br, I), are abundantly known[^31^, ^32^, ^33^, ^34^, ^35^, ^36^, ^37^, ^38^] and have found applications in organic synthesis.[^38^, ^39^]

In the present work we show that N‐heterocyclic carbene‐stabilized complexes of SF_2_, SeF_2_, SeF_4_ and TeF_4_ can be obtained as crystalline solids by the oxidation of the imidazol‐2‐thione, imidazol‐2‐selenone and imidazol‐2‐tellurone derivatives using xenon difluoride. Thus, the reaction of IPrE (E=S (**1S**),^[40]^ Se (**1Se**)^[41]^ and Te (**1Te**),^[42]^ IPr=1,3‐bis(2,6‐diisopropylphenyl)imidazol‐2‐ylidene) with one or two equivalents of xenon difluoride afforded the chalcogen(II) fluoride complexes IPrSF_2_ (**2S**, Figure 1) and IPrSeF_2_ (**2Se**) as well as the chalcogen(IV) fluoride complexes IPrSeF_4_ (**3Se**, Figure 2) and [IPr_2_TeF_3_][TeF_5_] (**3Te**, Figure 3). The latter eventually converted over the course of ca. 6 days into the mesoionic complex aIPrTeF_4_ (**4Te**, aIPr=1,3‐bis(2,6‐diisopropylphenyl)imidazol‐4‐ylidene) following a normal to abnormal coordination switch (Figure 3).^[30]^ This transformation is accelerated at higher temperatures and can reach completion at 40 °C within 3 days. Attempts to isolate a tellurium(II) fluoride complex, IPrTeF_2_, by reacting **1Te** with one equivalent of XeF_2_ were unsuccessful. Regardless of the stoichiometric ratio between the IPrTe and XeF_2_, **3Te** was in every case the major product initially forming, in the presence of several other minor side‐products. The reaction of free carbene IPr with one equivalent of TeF_4_ gave systematically **3Te** in a better purity. The opposite was true in the reaction between IPr and SeF_4_ which gave **3Se** with a significantly lower purity and yield compared with the oxidation reaction. While investigating methods of purification for **3Se** we isolated a few crystals that proved to be [IPrF][SeF_5_] (**4Se**). We determined that this species does not form by the reaction of **2Se** and excess XeF_2_ but can be rationally obtained by heating **3Se** at 80 °C in THF. A stable sulphur tetrafluoride complex, IPrSF_4_, could not be obtained by further oxidation of **2S** with XeF_2_ or by reaction of IPr with SF_4_. In solution, all complexes were extremely sensitive to adventitious traces of water and their further purification from traces of [IPrH]^+^ salts or IPrE proved to be frustratingly difficult despite arduous efforts. We speculate also that at least some of these complexes might be able to react to some extent with the borosilicate glass. In solid state however, the compounds seemed to be stable when stored under argon atmosphere at room temperature for at least several weeks.

**Figure 1:**
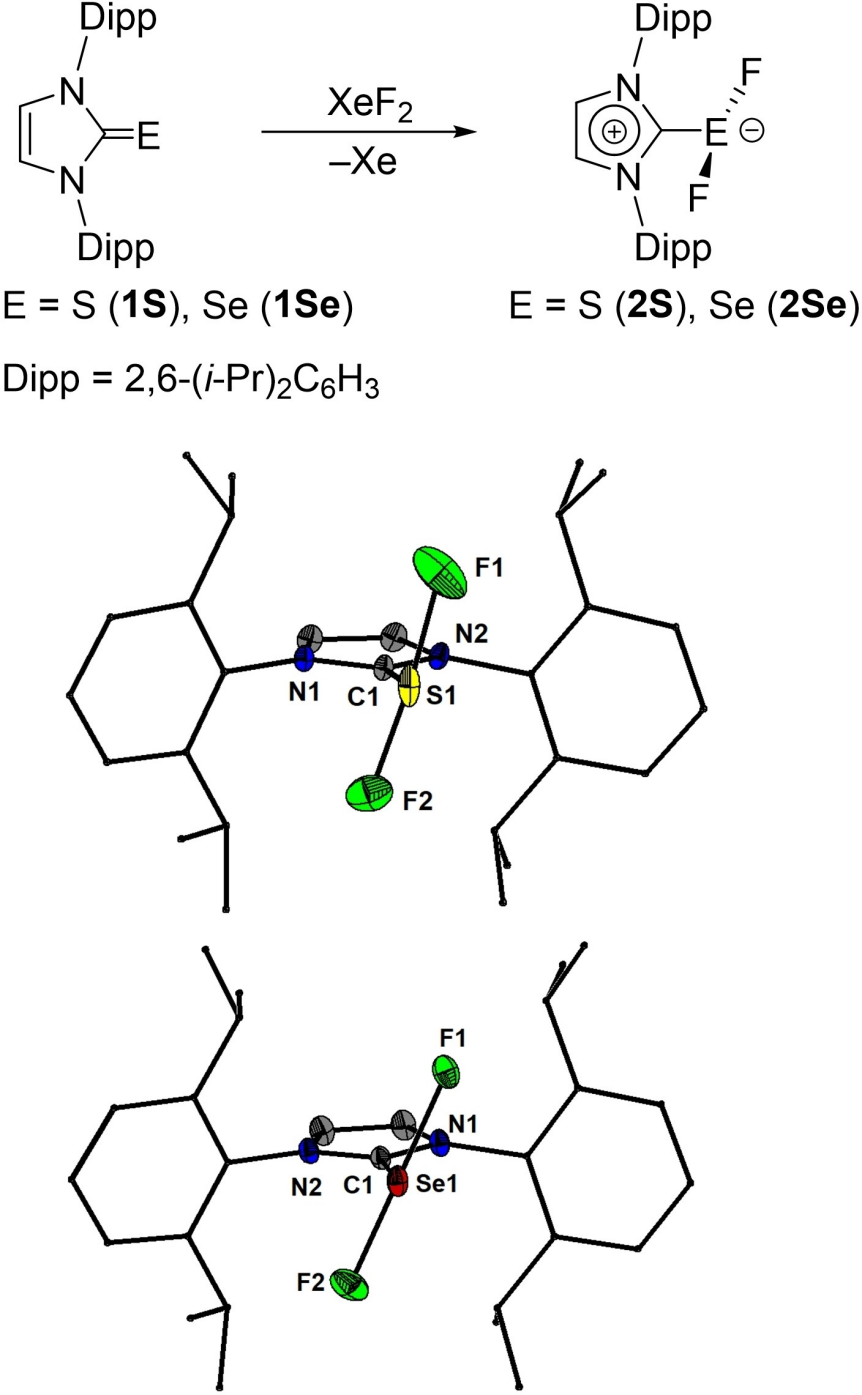
Synthesis of **2S** and **2Se** and molecular structures of **2S** and **2Se** showing 50 % probability ellipsoids and the numbering scheme of selected atoms. Selected bond lengths [Å] and angles [°] for **2S**: S1−C1 1.7298(16), S2−C4 1.7321(15), S1−F1 1.8261(16), S1−F2 1.8188(14), F1−S1−F2 170.98(7), F1−S1−C1 86.05(7), F2−S1−C1 85.40(7); for **2Se**: Se1−C1 1.8877(13), Se1−F1 1.9588(9), Se1−F2 1.9122(9), F2−Se1 F1 168.83(4), C1−Se1−F1 82.89(5), C1−Se1−F2 85.99(5).

**Figure 2:**
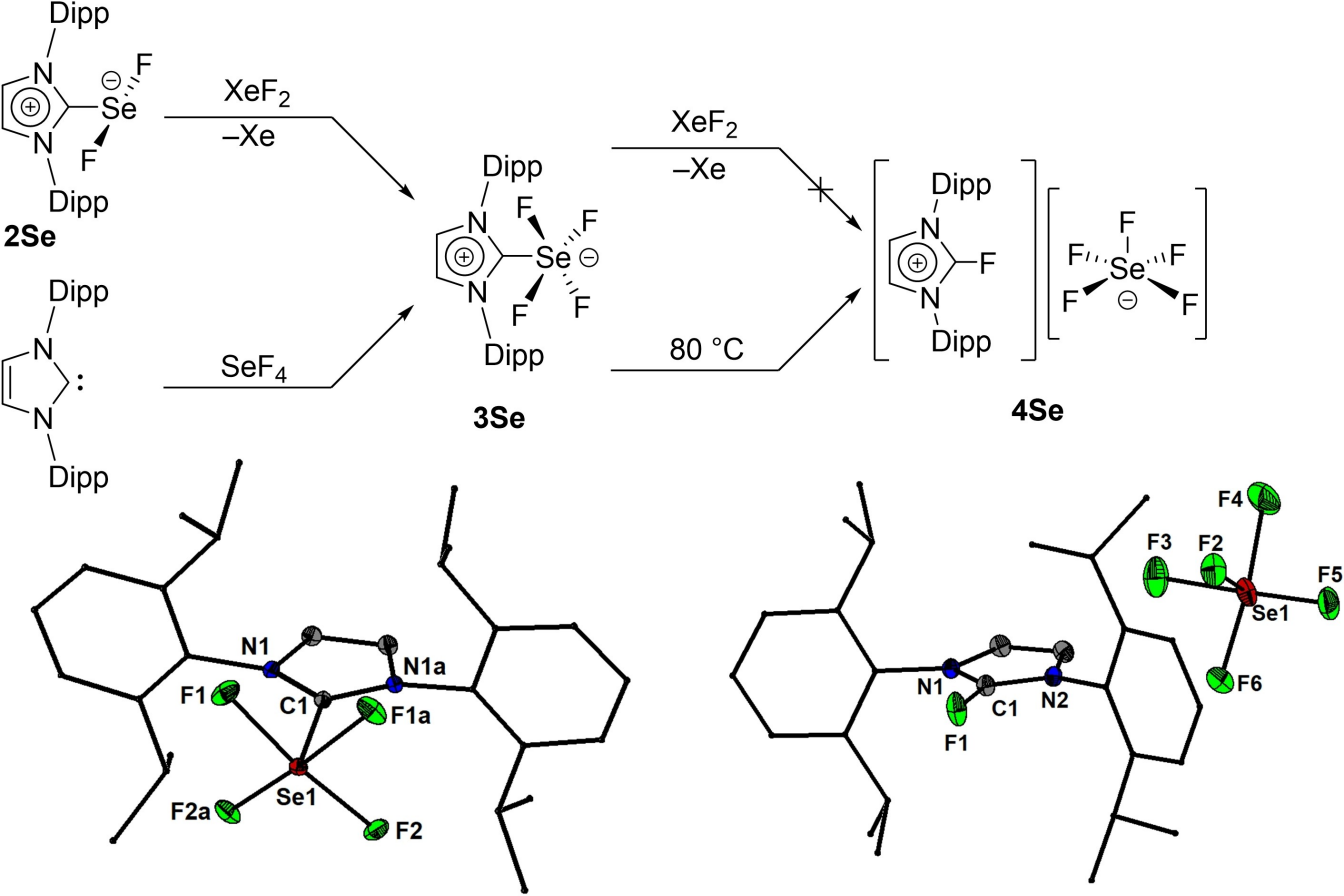
Synthesis of **3Se** and its decomposition to **4Se** and molecular structures of **3Se** and **4Se** showing 50 % probability ellipsoids and the numbering scheme of selected atoms. Selected bond lengths [Å] and angles [°] for **3Se**: Se1−F1 1.8281(7), Se1−F2 1.8675(7), Se1−C1 1.9668(15), F1−Se1−F2 168.92(3), F1−Se1−F2 168.92(3), F1−Se1−C1 85.62(4), F1−Se1−F1a 89.92(5); **4Se**: Se1−F5 1.8123(10), Se1−F2 1.7137(10), Se1−F3 1.8346(10), Se1−F6 1.8307(11), Se1−F4 1.8186(13), F5−Se1−F3 167.93(5), F5−Se1−F6 89.91(5), F2−Se1−F5 84.71(5), F2−Se1−F3 83.25(5), F2−Se1−F4 83.18(7), F4−Se1−F3 89.93(6), F4−Se1−F6 166.51(7).

**Figure 3:**
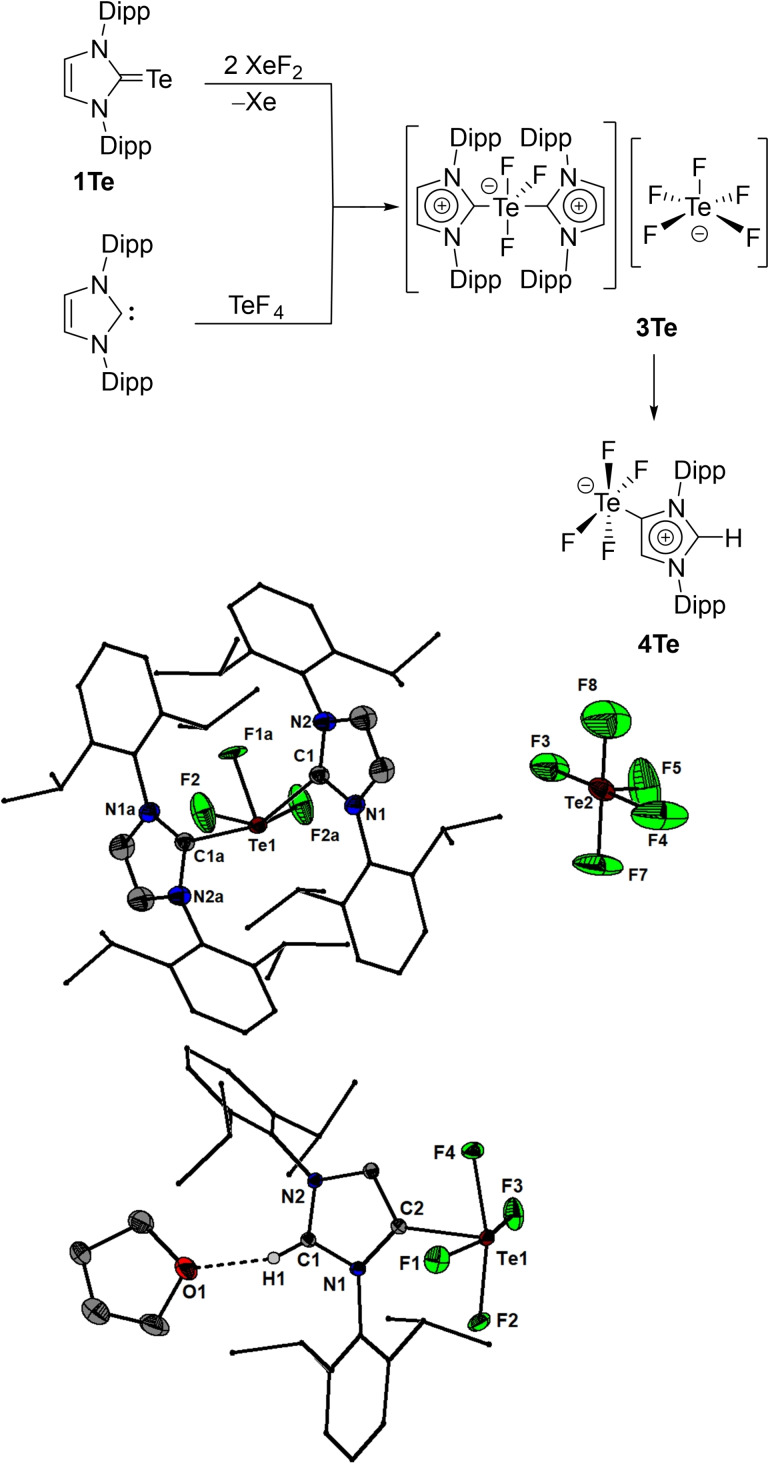
Synthesis of **3Te** and **4Te** and molecular structures of **3Te** and **4Te**⋅THF showing 50 % probability ellipsoids and the numbering scheme of selected atoms. Selected bond lengths [Å] and angles [°] for **3Te**: Te1−C1 2.2955(11), Te1−C1a 2.2883(11), Te1−F2 1.9726(10), Te1−F2a 1.9694(10), Te1−F1a 1.8616(14), C1a−Te1−C1 158.331(12), F2−Te1−F2a 154.750(14), F2−Te1−C1 89.03(4), F2a−Te1−C1 86.18(4), F1a−Te1−F2 103.25(12), F1a−Te1−F2a 77.14(7); for **4Te**⋅THF: Te1−F3 1.9875(8), Te1−F1 1.9568(9), Te1−F4 1.9758(9), Te1−F2 1.9786(10), Te1−C2 2.1169(12), F3−Te1 C2 80.60(4), F1−Te1−F3 165.35(4), F1−Te1−F4 88.50(4), F1−Te1−F2 89.18(5), F1−Te1−C2 84.79(4), F4−Te1−F3 88.43(4), F4−Te1−F2 163.95(4), F4−Te1−C2 82.06(4), F2−Te1−F3 89.82(5), F2−Te1−C2 81.92(4).

The ^19^F NMR spectra of **2S**, **2Se** and **3Se** displayed singlet resonance signals at *δ*=−97.1, −157.8 and 3.6 ppm, respectively, signals that were accompanied, in the case of **2Se** and **3Se**, by ^77^Se satellites (1136 and 255 Hz, respectively) matching the coupling constants observed for the multiplets assigned in the ^77^Se NMR spectra to these species: a triplet resonance at *δ*=1003.5 ppm for **2Se** and a quintet at *δ*=884.7 ppm for **3Se**. Remarkably, the ^19^F resonance reported for (I^*i*^Pr_2_Me_2_)SF_2_ (37.7 ppm)^[31]^ is at a significantly higher chemical shift compared to **2S**. Because of this marked difference and because we became motivated to determine the molecular structure of (I^*i*^Pr_2_Me_2_)SF_2_, which was not reported in the original study, we decided to reinvestigate this compound. Following closely the original procedure of reacting (I^*i*^Pr_2_Me_2_)SCl_2_ with AgF,^[31]^ we were, unfortunately, unable to isolate (I^*i*^Pr_2_Me_2_)SF_2_. Although we observed a very weak signal around 37.9 ppm by ^19^F NMR, the ^1^H NMR spectrum showed the major species to be (I^*i*^Pr_2_Me_2_)S. Attempting to oxidize (I^*i*^Pr_2_Me_2_)S with XeF_2_ failed as well to give the desired complex, leading us to assume that (I^*i*^Pr_2_Me_2_)SF_2_ might be too unstable to be isolated. The formation of **3Te** was confirmed by ^19^F NMR spectra which displayed a triplet resonance assigned to the apical fluorine atom (δ=−24.1 ppm) and a doublet resonance signal (δ=−91.5 ppm) for the remaining two basal fluorine atoms of [IPr_2_TeF_3_]^+^, as well as resonances assigned to the [TeF_5_]^−^ counter ion (quintet at δ=−31.8 ppm, apical F; doublet −37.5 ppm, basal F), thus confirming the retention of the solid state structure of **3Te** in solution. The ^125^Te NMR spectrum showed multiplet resonance signals corresponding to the cation (doublet of triplets at *δ*=1078.9 ppm) and the anion (doublet of quintets at *δ*=1144.4 ppm) with ^19^F−^125^Te coupling constants matching the ^125^Te satellites observed for the assigned ^19^F resonances. A slow conversion of **3Te** into the abnormal complex **4Te** (characterized by a singlet resonance signal at −47.0 ppm in the ^19^F spectrum and a quintet at 1202.5 ppm in the ^125^Te NMR) was observed. The observation of coupling between ^19^F−^125^Te (239 Hz) indicates that the molecular structure of **4Te** does not undergo conformational change in solution (at room temperature) as observed previously for R_3_POTeF_4_ (R=Me, Ph) and remains similar to the solid‐state structure.^[17]^

Single crystal X‐ray diffraction investigations revealed that in solid state **2S**, **2Se**, **3Se**, **3Te** and **4Te** are distinct monomers.^[43]^ In **2S** and **2Se** the coordination geometry around the chalcogen is a distorted T‐shape, while **3Se**, **3Te** and **4Te** feature distorted square pyramidal geometry arrangements around the chalcogen. The carbene occupies the apical positions in **3Se** and **4Te**. In the cationic **3Te** the carbene ligands coordinate trans with respect to each other in the basal plane while the apical position is occupied by a fluoride. The averaged S−F (1.819(1) Å) and Se−F (1.935(1) Å) bond distances in **2S** and **2Se** are significantly larger than the values determined by microwave spectroscopy for SF_2_ in the gas phase (1.589 Å)^[44]^ and SeF_2_ in an argon matrix (1.725 Å).^[27]^ The F−E−F angles in both **2S** (average 171.50(7)°) and **2Se** (average 168.84(4)°) deviate from the ideal 180° value as a result of electrostatic repulsion exerted by the lone pairs at the chalcogen. The average C−E bond lengths of 1.731(2) Å (**2S**) and 1.889(1) Å (**2Se**) are longer than the ^IPr^C=S (1.670(3) Å) and the ^IPr^C=Se bonds (1.827(6) Å)^[45]^ and are closer to the values observed generally for single C−S (e. g., 1.763(1) Å)^[46]^ or C−Se (e. g., 1.927(3) Å)^[47]^ bonds in diaryl sulfides and selenides. The Se(IV) complex **3Se** features shorter Se−F bond lengths (1.8675(7) and 1.8281(7) Å) and a more elongated C−Se bond length (1.967(2) Å) compared with **2Se** but retains a deviation of the diagonal F−Se−F bond angles (168.92(3)°). The Se−F bond lengths observed in **3Se** are comparable with those observed for the Se−F bonds contained in the basal plane of the [SeF_5_]^−^ anion of **4Se**, the thermal decomposition product of **3Se**, which has a similar coordination geometry around selenium. This anion features, however, a shortened bond distance between the apical fluoride and selenium (1.714(1) Å). In the cation of **3Te**, two different Te−F bond lengths can be observed depending on whether the F atom occupies the apical (1.862(1) Å) or basal (1.973(1) Å) positions and are similar to those observed in the R_3_POTeF_4_ (R=Me, Ph) complexes.^[17]^ The C−Te bonds (2.288(1) Å) are much more elongated compared to **1Te** (2.055(3) Å).^[45]^ The [TeF_5_]^−^ counter anion,^[24]^ which has the Te atom in a square pyramidal coordination geometry, was affected by significant positional disorder. The four fluorides in **4Te** are disposed in the basal plane. The Te−F bond lengths (average 1.973(1) Å) are comparable to those seen in the cation of **3Te** for the basal Te−F bonds, but the C−Te bond is significantly shorter (2.117(1) Å). The diagonal F−Te−F bonds deviate slightly from collinearity (165.35(4)° and 163.95(4)°).

The bonding within the donor acceptor complexes IPrEF_2_, IPrEF_4_, [IPr_2_EF_3_]^+^ and aIPrEF_4_ (E=S, Se, Te) was analysed by detailed density functional theory (DFT) calculations,^[48]^ energetically by energy decomposition analyses (EDA)^[49]^ and other energy descriptors derived from DFT, and electronically by a set of real‐space bonding indicators (RSBI). The overall or instantaneous interaction energy (E_int_) between the NHC and chalcogen fluoride fragments ranges from 330–520 kJ mol^−1^. In all cases, the estimated reorganization energy (E_re_; _ca._ 190–270 kJ mol^−1^) is larger than the estimated bond dissociation energy (E_d_; ca. 70–260 kJ mol^−1^), pointing towards a high energy expense upon complex formation caused by the need of structural reorganization of the chalcogen fluoride fragments. The E_int_ value is higher for S containing complexes than for their Se or Te analogues, which is most likely due to stronger primary S−C bonds, especially reflected in larger orbital attraction (E_orb_) values, pointing out bond covalency (Table S5). In S−C bonds, covalent bonding aspects dominate (slightly) over non‐covalent bonding aspects, i. e., the electrostatic attraction (E_elstat_), thus E_orb_>E_elstat_, whereas the opposite is observed for the Se−C and Te−C bonds (E_orb_<E_elstat_). This is supported by RSBI analysis (see below). Despite higher values for Pauli repulsion for EF_2_ than for EF_4_ containing complexes, E_int_ is nevertheless higher for the former as a consequence of higher electrostatic attraction and significantly higher orbital attraction. Including dispersion correction similarly increases Pauli repulsion (E_Pauli_), E_elstat_, E_orb_ and consequently also E_int_, as the two increased attractive forces overcompensate the increased Pauli repulsion. Notably, E_int_ and reorganization energies (E_re_) decrease for all compounds in the order S>Se>Te (Table S5 and S6), but the fragment sum (E_fs_) behaves different, as it shows no clear trend for EF_2_ complexes but increases significantly for EF_4_ complexes according to S<Se<Te. For **2Se** and **3Se**, the primary E−F and E−C bonds as well as numerous secondary F⋅⋅⋅C and F⋅⋅⋅H/Cπ interactions are reflected in the AIM (Figure 4a and e)^[50]^ bond topologies and the NCI *iso*‐surfaces (Figure 4b and f).^[51]^ The highly polar E−F bonds are characterized by electron density (ED, ρ(**r**)) values at the bond critical point (bcp) of 0.8–1.5 eÅ^−3^ for S, 0.9–1.3 eÅ^−3^ for Se, and 0.6–1.0 eÅ^−3^ for Te (Table S7). With few exceptions, bond ellipticities (ϵ) are below 0.15, indicating directed bonds with low electron smearing. Particularly high kinetic energy density over ED ratios (G/ρ(**r**)) larger than 1 point towards dominant ionic bond contributions, whereas equally high (negative) values for the total energy density over ED ratio (H/ρ(**r**)) indicate dominant covalent bond contributions. For the S−F bonds, G/ρ(**r**) and H/ρ(**r**) are typically large and similar, indicating the high relevance of both bonding aspects and resulting in a Laplacian of the ED (∇^2^ρ(**r**)) close to zero. For the Se−F and Te−F bonds, non‐covalent bonding aspects start to dominate over covalent bonding aspects (G/ρ(**r**) > |H/ρ(**r**)|, clearly positive ∇^2^ρ(**r**) value). Accordingly, only for the strong and short S−F contacts corresponding S−F bonding basins are formed in the ELI−D as consequence of covalent bond aspects, although being very small (V_ELI_=0.1–0.7 Å^3^) and little populated (N_ELI_=0.1–0.8 e). The less polar S−C and Se−C bonds are dominated by covalent bonding aspects (G/ρ(**r**) < |H/ρ(**r**)|, clearly negative ∇^2^ρ(**r**) value), whereas both bonding aspects are balanced in the Te−C bonds. Complex formation transforms the lone‐pair basin of the carbene C atom into a bonding E−C basin. In this course, the basin volumes (V_ELI_) shrinks from 15.1 Å to 3.7–9.5 Å^3^ and the localizabilities from 2.53 to 1.83–2.02, indicating increased electron sharing. This is further reflected in the Raub‐Jansen index (RJI),^[52]^ which is a measure for bond polarity as it quantifies the relative electron populations within an ELI−D basin being located in adjacent (typically bonding) AIM atomic basins. The RJI approaches 50 % for homopolar covalent bonds and becomes larger than 90 % for high polar dative and ionic bonds. The intermediate regime is occupied by polar‐covalent interactions, such as the E−C bonds (Table S8). Particularly low RJI values are obtained for regular and abnormal complexes, IPrEF_4_ and aIPrEF_4_. The electron populations (N_ELI_), however, are little affected, ranging from 2.2–2.6 in the complexes compared to 2.44 in the free carbene. Notably, they are decreasing for the IPrEF_2_ and abnormal complexes aIPrEF_4_, but increasing for IPrEF_4_ and [IPr_2_EF_3_]^+^. The secondary F⋅⋅⋅H/C_π_ interactions in all complexes are weak non‐covalent forces, which is reflected in low ED values typically below 0.1 eÅ^−3^, G/ρ(**r**) being larger than 0.75 a.u. and H/ρ(**r**) being positive. In NCI, extended greenish to bluish areas are observed (Figure 4b and f).^[51]^ Most E⋅⋅⋅H/C_π_ bond paths are curved and the bond ellipticities can become as large as 4.4, which is typical for such weak interactions, and results in AIM bond path lengths (d1+d2, d1 and d2 are the distance from atom 1 or 2 to the bcp) larger than the geometric atom‐atom distance. Although each of these interactions are apparently weak, the sum of them significantly contributes to the stabilization energy between the carbene and the EF_4_ fragment. For **2Se** and **3Se**, *iso*‐surface representations of the ELI−D are displayed in Figure 4c and g.^[53]^ For clarity, the non‐bonding lone‐pair basins (LP(E)) and bonding E−C basins are highlighted in solid green in the complexes. For EF_2_, adduct formation has a considerable effect on the ELI−D LP basin volumes, which are decreased by 30–50 % (Table S9). This is accompanied by lower electron localizabilities at the attractor position of the LP basins by about 0.3, indicating more pronounced electron sharing with the environment. This is, however, *not* accompanied by considerable changes in the basin populations, which are between 2.2 and 2.3 e in all cases. To the contrary, EF_4_ shows a somewhat unpredictable behaviour: only slightly decreased LP basin volumes in the regular carbene complexes, but drastically enlarged volumes in the abnormal complexes aIPrEF_4_, electron localizabilities even increased in some cases, but basin populations varying only to a small extent. With a grid‐step size of 0.05 bohr, these results should not be caused by an unsuitable integration routine, but are most likely due to different structural motifs, especially in the abnormal complexes aIPrEF_4_, whereas the two F−S−F planes are about 45° to the central ring plane in the regular carbene structure, one F−Se−F or F−Te−F plane is almost coplanar (the other one almost perpendicular) in the abnormal carbene complexes. The ELI−D E−C bonding basins of the EF_2_ adducts show strongly enhanced electron localizabilities in direction of the LP basins (bluish areas), indicating electronic interactions between these two basin types (Figure 4d). In **2S**, the E−C and LP basins are still fused at an *iso*‐value of 1.3 (Figure S22c). The pear‐shaped E−C bonding basins of the EF_4_ adducts, however, suggest only weak electronic non‐localized interactions to the S−F bonding or LP(F) basins, visible as ring‐shaped greenish to bluish gradients (Figure 4h and Figures S24–S28). The cationic complexes [IPr_2_EF_3_]^+^ show both basin interaction types, as they carry three F atoms as well as one lone‐pair in the central EF_3_(LP) plane (Figures S29d–S31d).

**Figure 4:**
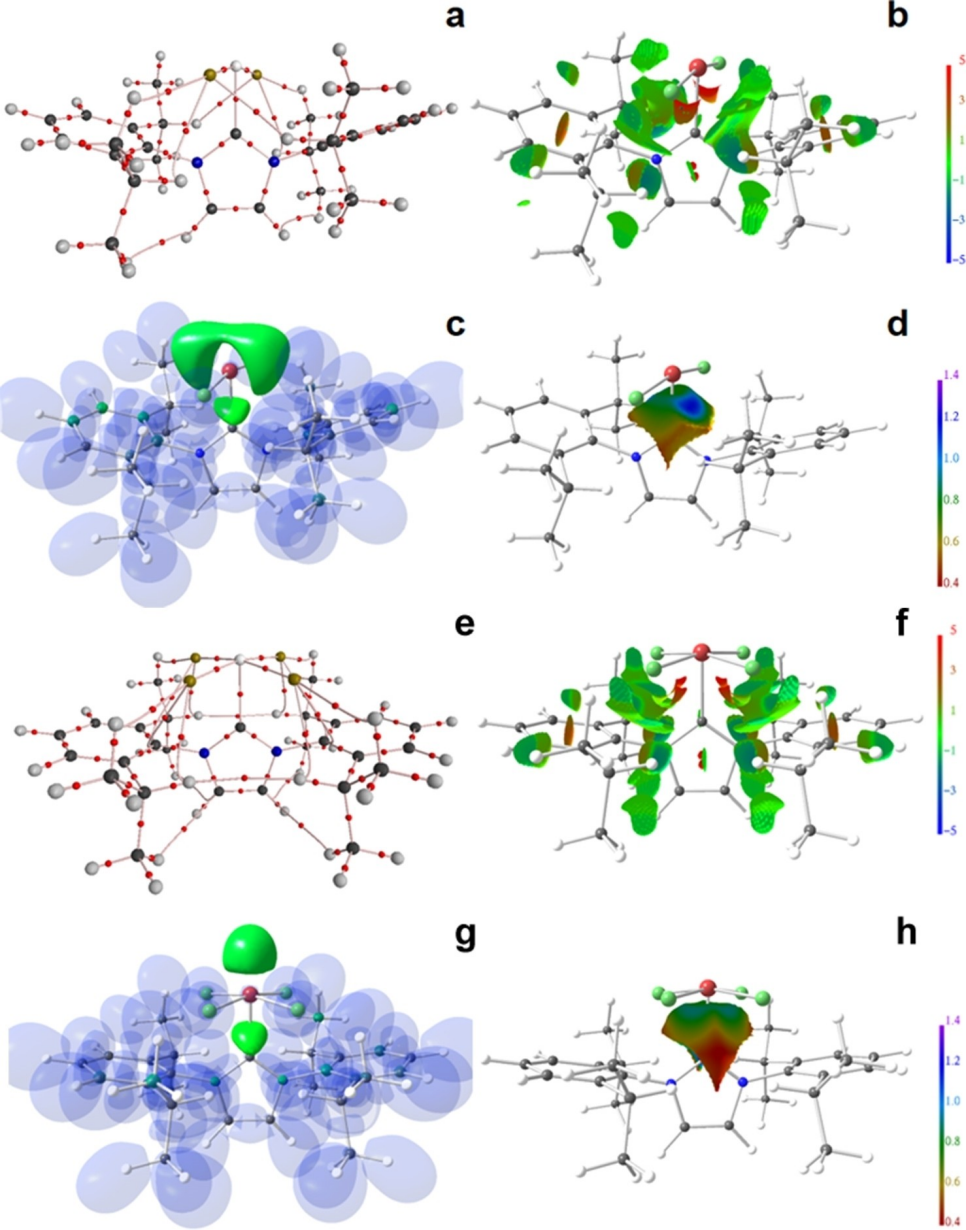
RSBI analysis of **2Se** and **3Se** respectively: (a) and (e) AIM bond paths motif, (b) and (f) NCI *iso*‐surface at s(**r**)=0.5, (c) and (g) ELI−D localization domain representation at *iso*‐value of 1.3, (d) and (h) ELI−D distribution mapped on the E−C ELI−D bonding basin.

In summary, the carbene complexes IPrSF_2_ (**2S**) and IPrSeF_2_ (**2Se**) and IPrSeF_4_ (**3Se**) were obtained as crystalline materials without the need to generate the highly unstable and toxic parent chalcogen fluorides (IPr=1,3‐bis(2,6‐diisopropylphenyl)imidazol‐2‐ylidene). Extending this chemistry to tellurium led us to isolate a metastable ionic complex, [IPr_2_TeF_3_][TeF_5_] (**3Te**), which slowly converted into the abnormal NHC complex aIPrTeF_4_ (**4Te**). Attempts to obtain analogous NHC complexes of highly reactive SF_4_ or (the unknown) TeF_2_ (i. e., IPrSF_4_ and IPrTeF_2_) in a similar way were unsuccessful. The thermodynamic stability of the isolated complexes results from covalent and electrostatic contributions alike.

## Conflict of interest

The authors declare no conflict of interest.

## Supporting information

As a service to our authors and readers, this journal provides supporting information supplied by the authors. Such materials are peer reviewed and may be re‐organized for online delivery, but are not copy‐edited or typeset. Technical support issues arising from supporting information (other than missing files) should be addressed to the authors.

Supporting Information

## Data Availability

The data that support the findings of this study are available from the corresponding author upon reasonable request.
