# Supplementary material for: Donor Acceptor Complexes between the Chalcogen Fluorides SF2, SeF2, SeF4 and TeF4 and an N‐Heterocyclic Carbene
Source: Chemistry. 2022 Jun 20;28(45):e202201023. doi: 10.1002/chem.202201023 (PMC9544779; doi:10.1002/chem.202201023)
Supplement: Supplementary file 1 — Supporting Information [file CHEM-28-0-s001.pdf]

Chemistry–A European Journal

Supporting Information

Donor Acceptor Complexes between the Chalcogen Fluorides SF_2, SeF_2, SeF_4 and TeF_4 and an N-Heterocyclic Carbene

Pascal Komorr, Marian Olaru,* Emanuel Hupf, Stefan Mebs,* and Jens Beckmann*

Table of Contents

Experimental procedures 2

General information 2

Synthesis and characterization of $IPrSF_2$ (2S) 3

Synthesis and characterization of $IPrSeF_2$ (2Se) 6

Synthesis and characterization of $IPrSeF_4$ (3Se) 9

Synthesis of [IPrF][SeF_5] (4Se) 12

Synthesis and characterization of [IPr_2-TeF_3][TeF_5] (3Te) and [*a*IPr-TeF_4] (4Te) 13

Attempted Synthesis of ($I^iPr_2Me_2$)SF_2 18

Crystallographic data 22

Computational details. 25

References 45

Experimental procedures

General information

Unless otherwise stated, all reactions, manipulations, work-up and purifications were performed under inert argon atmosphere using anhydrous solvents. Reagents used in this work were obtained commercially and were used as received. The compounds IPrHCl[S1], IPr[S2], 1S[S3], 1Se[S4], 1Te[S5], (I^{i}Pr$_2$Me$_2$)S[S6] and (I^{i}Pr$_2$Me$_2$)SCl$_2$[S6] were prepared according to published procedures. Anhydrous solvents were collected from an SPS800 mBraun solvent purification system or distilled from K-benzophenone ketyl and stored over 3 Å molecular sieves. Deuterated solvents and 1,2-difluorobenzene were degassed and dried over 3 Å molecular sieves under argon.

Unless otherwise noted, NMR spectra were recorded at room temperature on Bruker Avance Neo 600 MHz spectrometers. ^{1}H, ^{13}C{^{1}H}, ^{19}F, ^{77}Se, ^{125}Te spectra are reported on the δ scale (ppm) and are referenced against $SiMe_4$, $CFCl_3$, Me_2Se and Me_2Te respectively. ^{1}H and ^{13}C{^{1}H} chemical shifts are reported relative to the residual peak of the solvent (C_6DH_5: 7.16 ppm, for C_6D_6; $CDHCl_2$: 5.32 ppm, for CD_2Cl_2; C_4D_7HO: 3.58 ppm for THF-d_8) in the ^{1}H NMR spectra, and to the peak of the deuterated solvent (C_6D_6: 128.06 ppm; CD_2Cl_2: 53.84 ppm; THF-*d8*: 67.21 ppm) in the ^{13}C{^{1}H} NMR spectra.[S7] The assignment of the ^{1}H and ^{13}C{^{1}H} resonance signals was made in accordance with the COSY, HSQC, HMBC and NOESY spectra.

Synthesis and characterization of $IPrSF_2$ (2S)

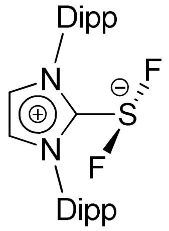

In a glove box, a screw cap septum vial was charged with **1S** (102 mg, 0.242 mmol), XeF_2 (44 mg, 0.26 mmol, 1.07 eq.) and a stirring bar. Cold THF (1 mL, −10 °C) was added while stirring, and the suspension was shaken for 1 min. The solvent was removed *in vacuo*, affording a colourless to light-yellow solid (101 mg). A yield of 85% (93 mg, 0.20 mmol) was estimated based on ^{1}H NMR data. The purity of the product was evaluated at 92-94% by NMR (6-8% of **1S** was identified as an impurity).

Alternatively, 1,2-difluorobenzene was used as a solvent and the reaction was also carried out under Schlenk-conditions, yielding similar results. A crystallisation as described for **2Se** and **3Se** did not improve the purity of the product.

^{1}H NMR (601 MHz, THF-d_8): δ = 7.71 (s, 2H, N–CH), 7.49 (t, J = 7.8 Hz, 2H, *p*-Ar), 7.34 (d, J = 7.8 Hz, 4H, *m*-Ar), 2.79 (hept, J = 6.8 Hz, 4H, CH–Me_2), 1.27 (d, J = 6.7 Hz, 12H, CH_3), 1.14 (d, J = 7.0 Hz, 12H, CH_3) ppm. **^{13}C{^{1}H} NMR** (151 MHz, THF-d_8): δ = 160.22 (C–S), 147.62 (*o*-Ar), 133.69 (*ipso*-Ar), 131.51 (*p*-Ar), 124.82 (N–CH), 124.10 (*m*-Ar), 29.80 (CH–Me_2), 25.86 (CH_3), 23.41 (CH_3) ppm. **^{19}F NMR** (565 MHz, THF-d_8): δ = −97.10 (s, br) ppm.

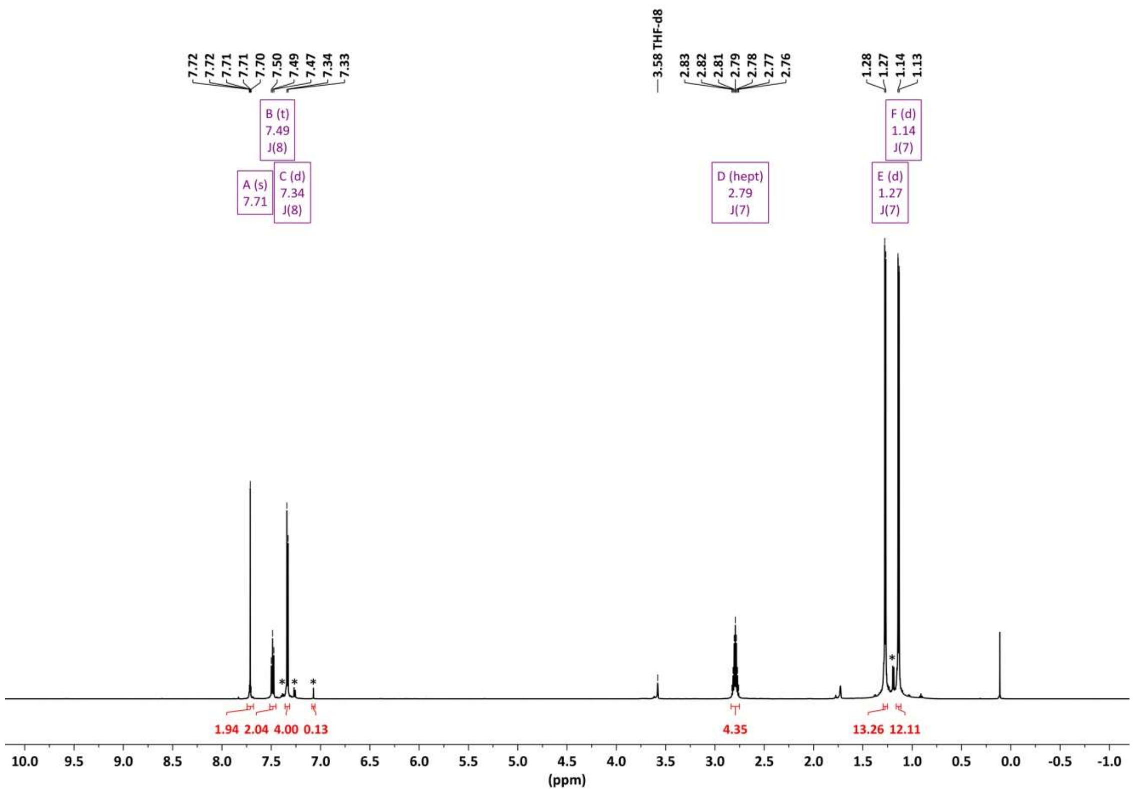

Figure S1: ^{1}H NMR (600 MHz, THF-d_8) spectrum of **2S** (* denotes **1S**).

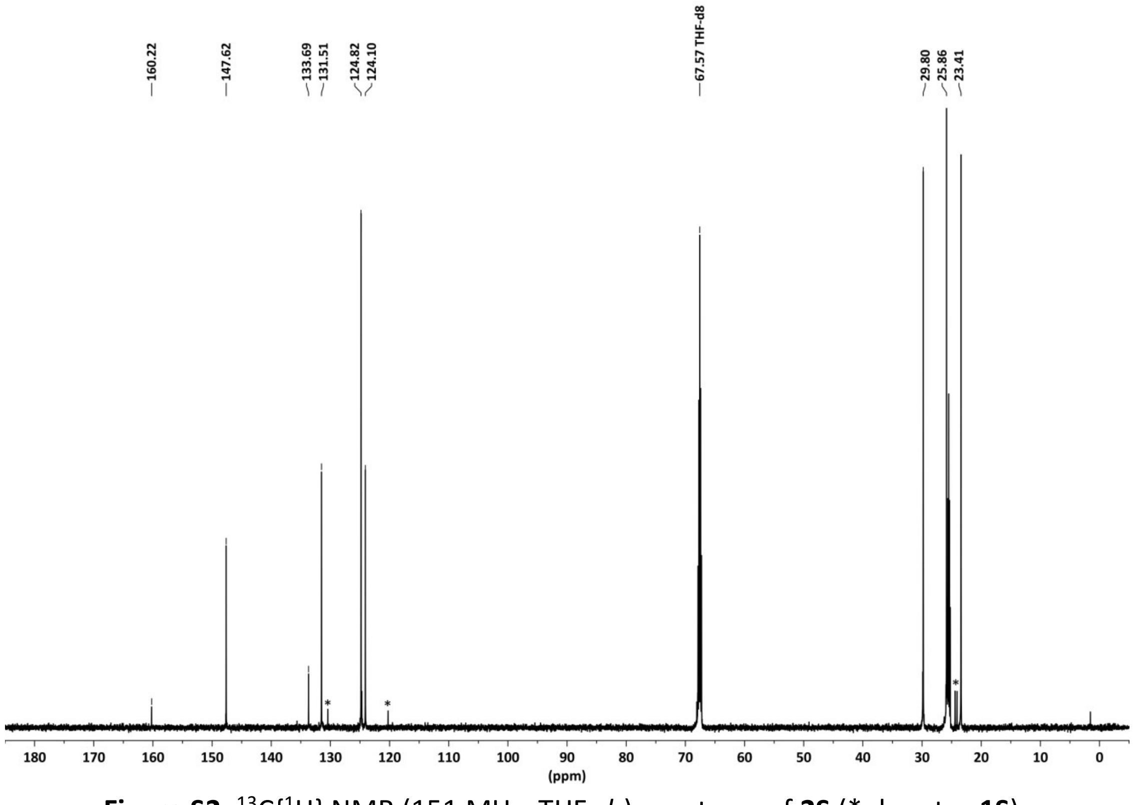

Figure S2. $^{13}C\{^{1}H\}$ NMR (151 MHz, THF-d_8) spectrum of **2S** (* denotes **1S**).

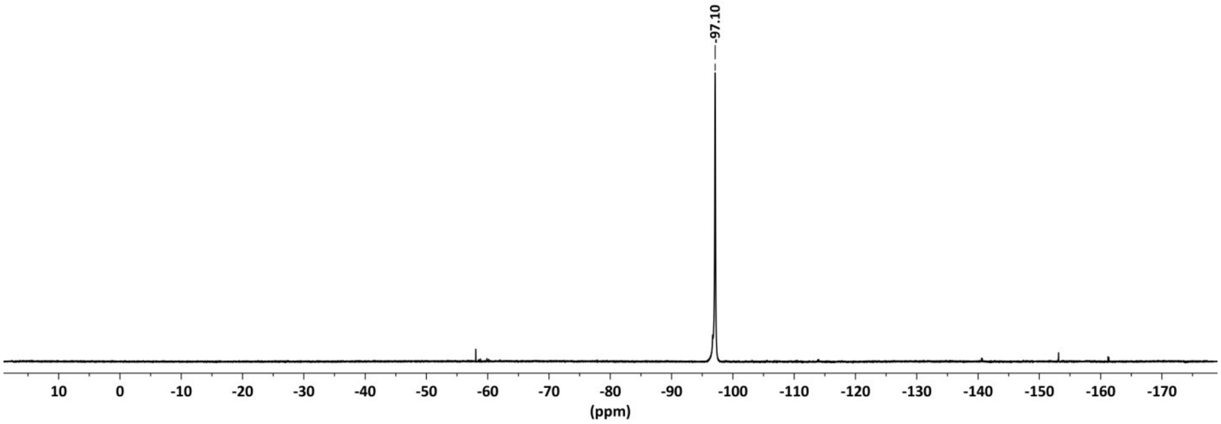

Figure S3. ^{19}F NMR (565 MHz, THF-d_8) spectrum of **2S.**

Synthesis and characterization of IPrSeF$_2$ (2Se)

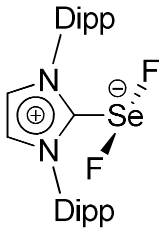

In a glovebox, a screw cap septum vial was charged with **1Se** (80.0 mg, 0.171 mmol), XeF$_2$ (1.1 eq., 32.0 mg, 0.188 mmol) and a stirring bar. While stirring, 1,2-difluorobenzene (2 mL, pre-cooled to −10 °C) was added and the vial was shaken until a clear solution was present. *n*-Hexane (6 mL) was immediately layered on top of the solution and the vial was left to crystallise at −10 °C. After 3 d the supernatant was decanted and the crystaline solid was dried *in vacuo* to obtain a light-yellow solid (68.8 mg). A yield of 75% (65 mg, 0.13 mmol) was estimated based on ^{1}H NMR data. The purity of the product was evaluated at 95% by NMR (5% of [IPrH][SeF$_5$] was identified as an impurity).

^{1}H NMR (601 MHz, THF-*d*$_8$): δ = 7.82 (s, 2H, N–CH), 7.49 (t, J = 7.8 Hz, 2H, *p*-Ar), 7.33 (d, J = 7.8 Hz, 4H, *m*-Ar), 2.76 (hept, J = 7.0 Hz, 4H, CH–Me$_2$), 1.28 (d, J = 6.7 Hz, 12H, CH$_3$), 1.13 (d, J = 7.0 Hz, 12H, CH$_3$) ppm.**^{13}C NMR** (151 MHz, THF-*d*$_8$): δ = 155.82 (C–Se), 147.57 (*o*-Ar), 134.29 (*ipso*-Ar), 131.61 (*p*-Ar), 125.42 (N–CH), 124.82 (*m*-Ar), 29.82 (CH–Me$_2$), 25.97 (CH$_3$), 23.37 (CH$_3$) ppm.**^{19}F NMR** (565 MHz, THF-*d*$_8$): δ = −157.83 (s, $^{1}J_{F\text{-}Se}$ = 1136 Hz) ppm.**^{77}Se NMR** (115 MHz, THF-*d*$_8$): δ = 1003.5 (t, $^{1}J_{Se\text{–}F}$ = 1134 Hz) ppm.

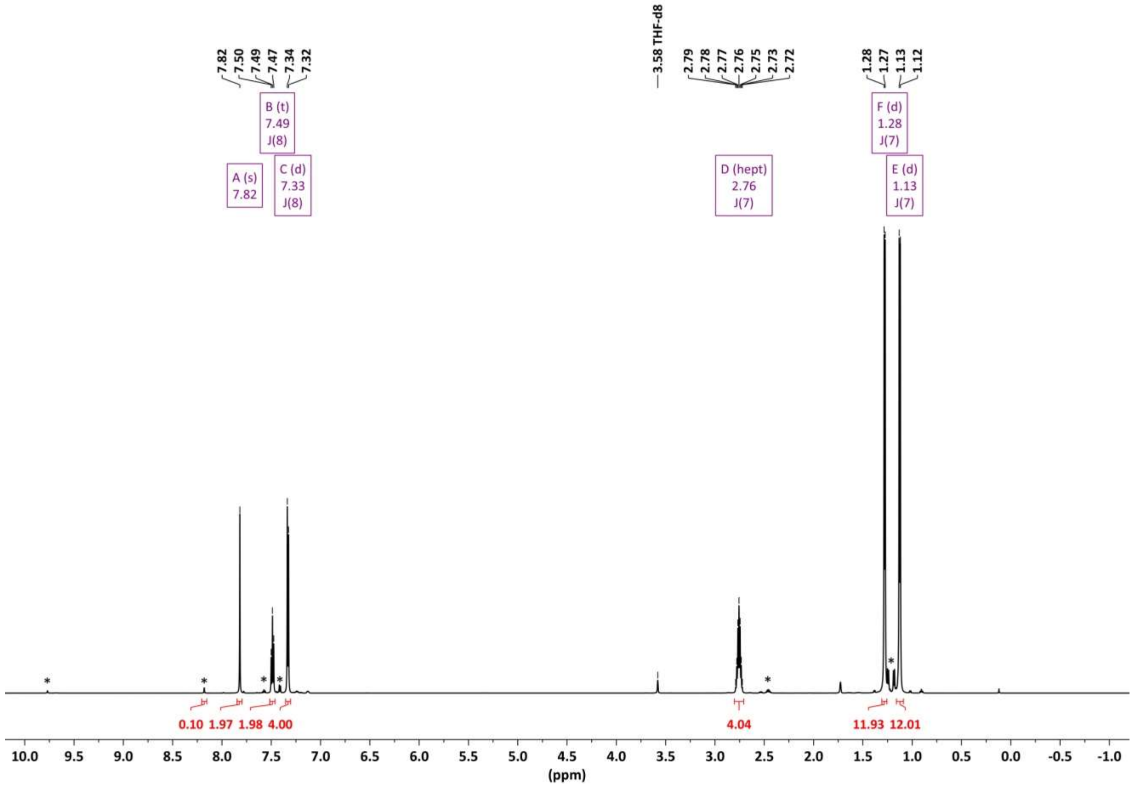

Figure S4: ^{1}H NMR (600 MHz, THF-d_8) spectrum of **2Se** (* denotes $[IPrH]^+$).

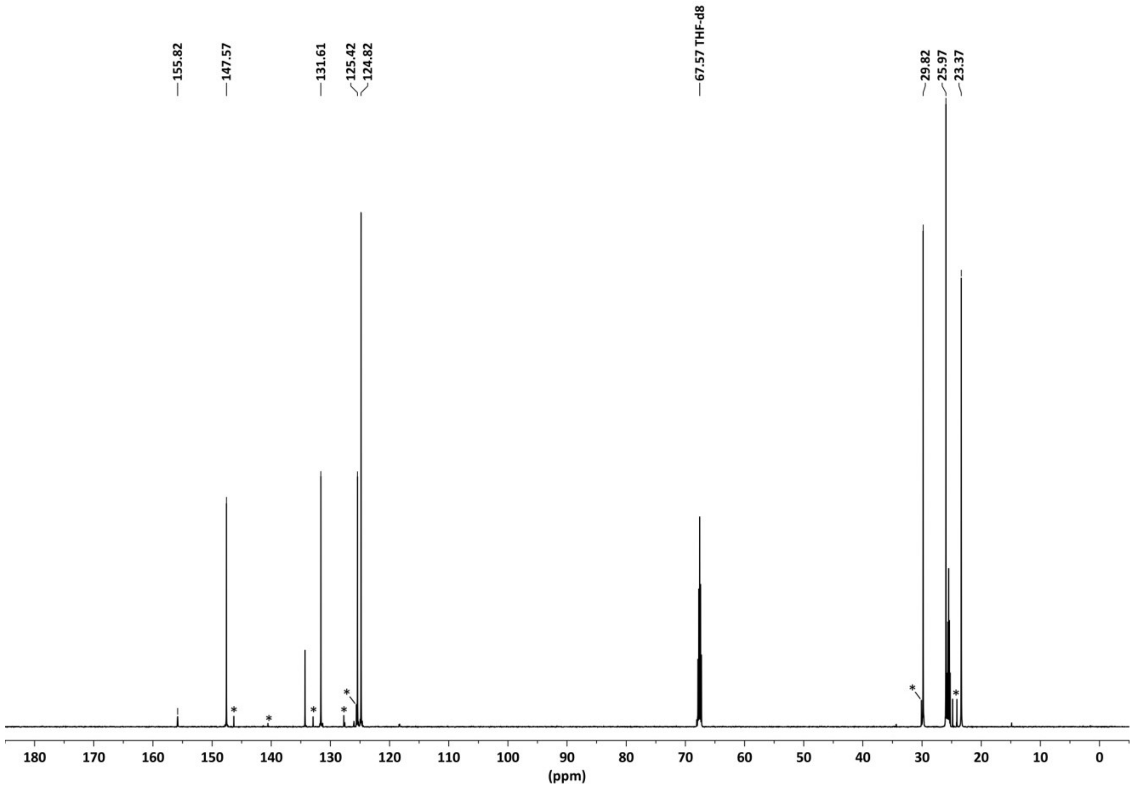

Figure S5: $^{13}C\{^{1}H\}$ NMR (151 MHz, THF-d_8) spectrum of **2Se** (* denotes $[IPrH]^+$).

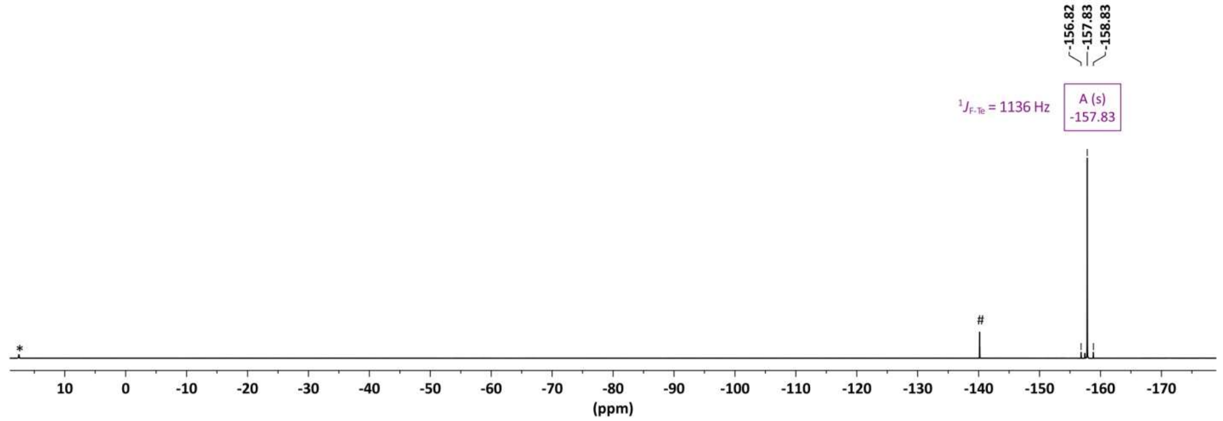

Figure S6: ^{19}F NMR (565 MHz, THF-d_8) of **2Se** (* denotes $[SeF_5]^-$, # denotes 1,2-difluorobenzene).

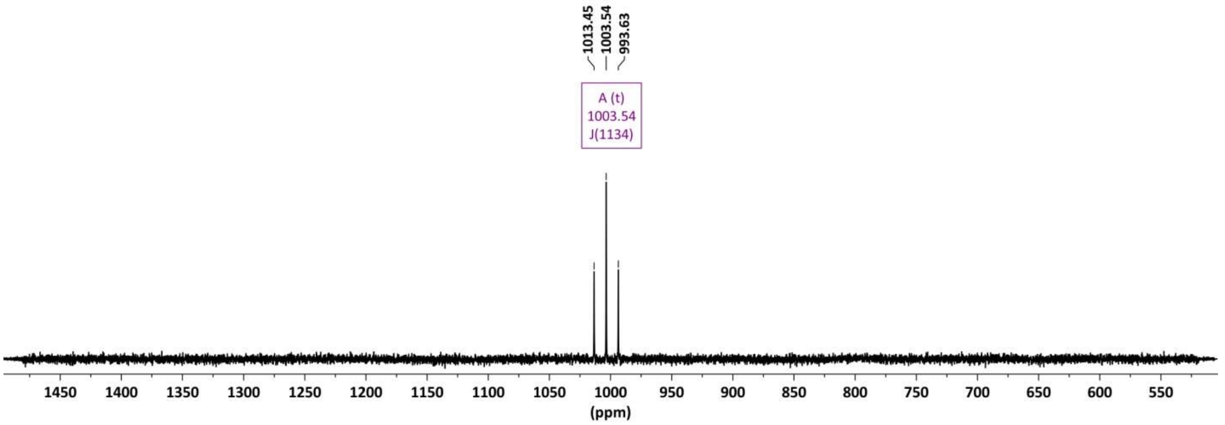

Figure S7: ^{77}Se NMR (115 MHz, THF-d_8) spectrum of **2Se.**

Synthesis and characterization of IPrSeF$_4$ (3Se)[*]

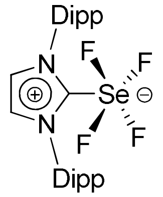

In a glovebox, a screw cap septum vial was charged with **1Se** (100 mg, 0.214 mmol), XeF$_2$ (43.0 mg, 0.372 mmol) and a stirring bar. While stirring, 1,2-difluorobenzene (4 mL, pre-cooled to −10 °C) was added and the vial was shaken until a clear solution was present. *n*-Hexane (8 mL) was immediately layered on top of the solution and the vial was left to crystallise at −10 °C. After 3 d the supernatant was decanted and the crystalline solid was dried *in vacuo* to obtain a light-yellow solid (111 mg). A yield of 86% (0.10 g, 0.18 mmol) was estimated based on ^{1}H NMR data. The purity of the product was evaluated at 90% by NMR ([IPrH][SeF$_5$] and an unidentified compound were present as impurities).

^{1}H NMR (601 MHz, THF-d_8): δ = 7.66 (s, 2H, N–CH), 7.41 (t, J = 7.5 Hz, 2H, *p*-Ar), 7.26 (d, J = 7.5 Hz, 4H, *m*-Ar), 2.70 (hept, J = 6.7 Hz, 4H, CH–Me$_2$), 1.35 (d, J = 6.1 Hz, 12H, CH$_3$), 1.11 (d, J = 6.6 Hz, 12H, CH$_3$) ppm. **^{13}C NMR** (151 MHz, THF-d_8): δ = 160.99 (C–Se), 146.80 (*o*-Ar), 136.75 (*ipso*-Ar), 130.88 (*p*-Ar), 126.24 (N–CH), 124.07 (*m*-Ar), 30.77 (CH–Me$_2$), 26.14 (CH$_3$), 22.93 (CH$_3$) ppm. **^{19}F NMR** (565 MHz, THF-d_8): δ = 3.63 (s, $^{1}J_{F\text{–}Se}$ = 255 Hz) ppm. **^{77}Se NMR** (115 MHz, THF-d_8): δ = 884.72 (p, $^{1}J_{Se\text{–}F}$ = 256 Hz) ppm.

[*] The following alternative synthesis method gave **3Se** with with a significantly lower purity: SeF$_4$ (0.1 mL, ca. 0.28 g, 1.8 mmol) was added over a precooled (–80 °C) solution of IPr (0.78 g, 2.0 mmol) in THF (12 mL). After stirring at this temperature for 10 minutes, the cooling bath was removed and the reaction mixture was brought to room temperature, where the resulting red-brown solution was layered with 12 mL of hexane. Yellow crystals (confirmed by XRD to be **3Se**) and a red-brown precipitate formed. Purification of **3Se** from this mixture was not successful.

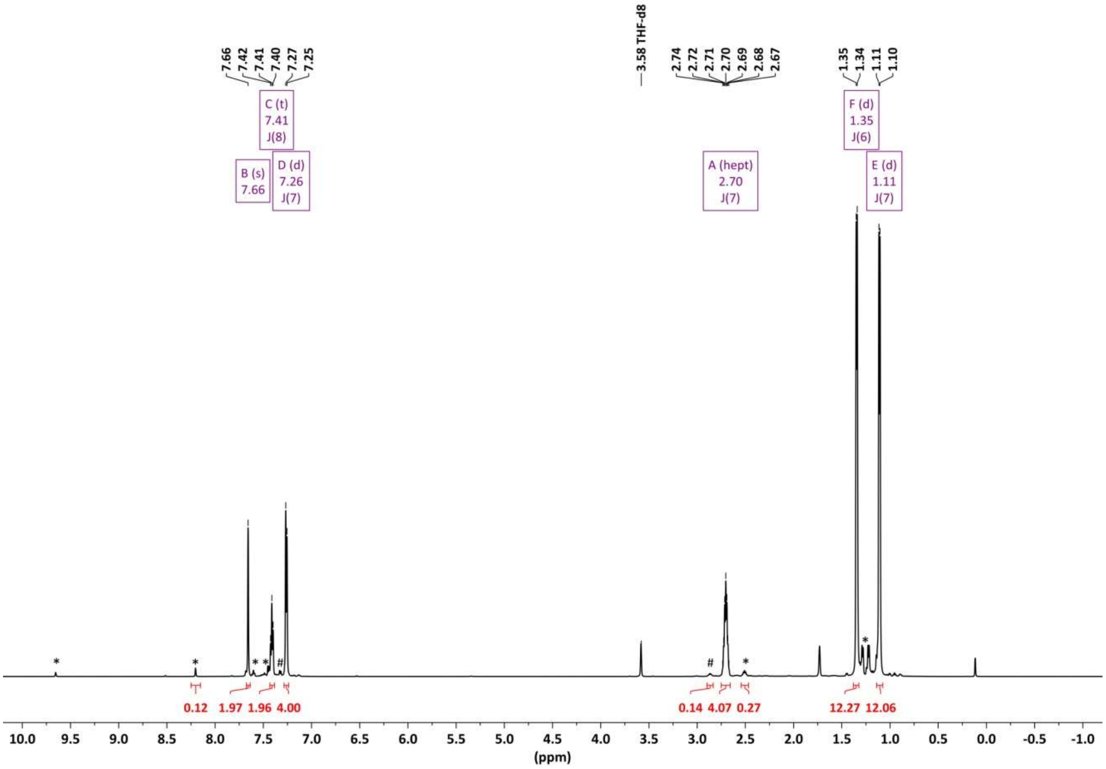

Figure S8: ^{1}H NMR (600 MHz, THF-d_8) spectrum of **3Se** (* denotes $[IPrH]^+$, $^{\#}$ denotes an unidentified side product).

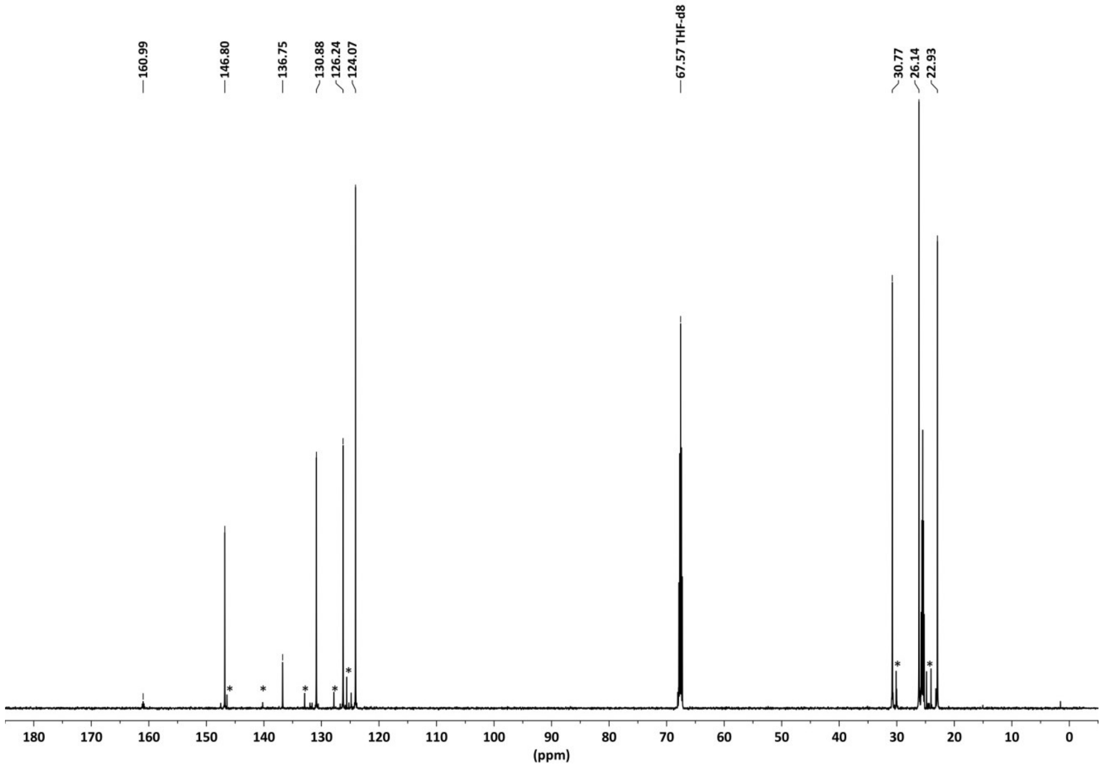

Figure S9: ^{13}C{^{1}H} NMR (151 MHz, THF-d_8) spectrum of **3Se** (* denotes $[IPrH]^+$).

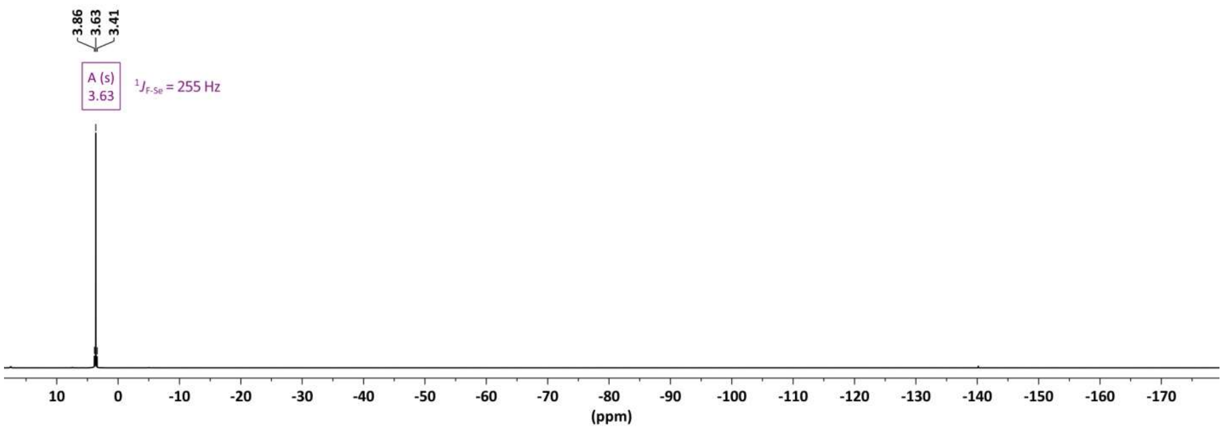

Figure S10: ^{19}F NMR (565 MHz, THF-d_8) of **3Se.**

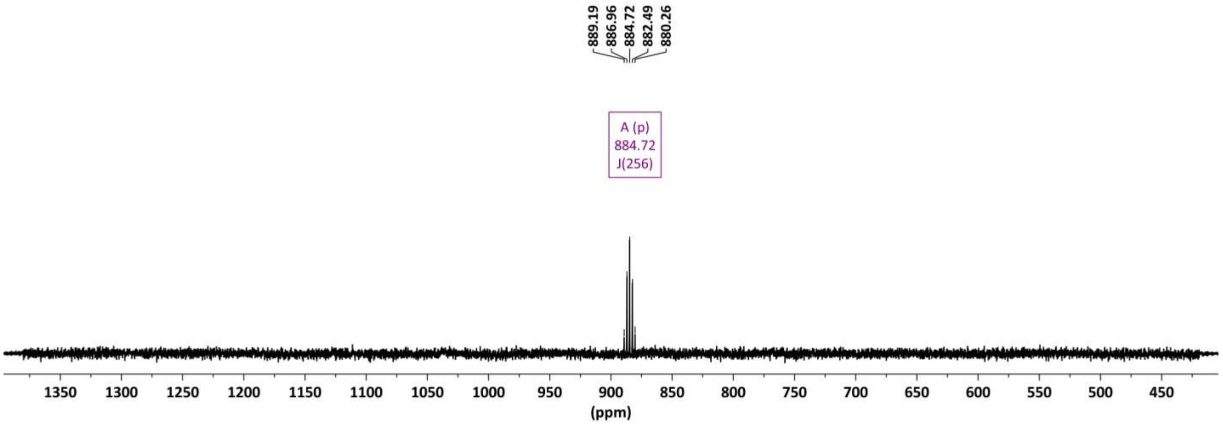

Figure S11: ^{77}Se NMR (115 MHz, THF-d_8) spectrum of **3Se.**

Synthesis of [IPrF][SeF_5] (4Se)

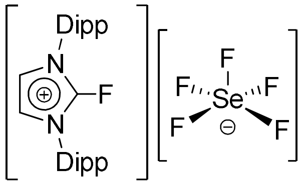

3Se (25 mg, 90% purity, 0.046 mmol) was placed in a J. Young NMR tube, dissolved in DCM-d_2 (0.5 mL) and heated to 60 °C for 3 d. NMR spectra revealed that [IPrF][SeF_5] was the main product. The resonances in ^{1}H, ^{13}C, ^{19}F and ^{77}Se mached closely the values reported in the literature for the [IPrF] cation[S8] and the [SeF_5] anion[S9]:

^{1}H NMR (600 MHz, CD_2Cl_2): δ = 7.82 (d, J = 2.3 Hz, 2H, N–C*H*), 7.69 (t, J = 7.9 Hz, 2H, *p*-Ar), 7.46 (d, J = 7.9 Hz, 4H, *m*-Ar), 2.49 (hept, J = 6.9 Hz, 4H, C*H*–Me_2), 1.34 (d, J = 6.7 Hz, 12H, CH_3), 1.22 (d, J = 7.0 Hz, 12H, CH_3) ppm. **^{13}C NMR** (151 MHz, CD_2Cl_2): δ = 146.03, 143.91 (d, J_{CF}= 280.4 Hz), 133.76, 125.97, 124.76, 122.41 (d, J_{CF} = 5.2 Hz), 30.17, 24.42, 23.94 ppm. **^{19}F NMR** (565 MHz, CD_2Cl_2, −30 °C): δ = 56.10 (s, br, J_{SeF} = 1127.5 Hz, 1F, SeF_{apical}), 14.95 (s, br, 4F, SeF_{basal}), -106.84 (s, J_{CF}= 278.7 Hz, J_{CF} = 563.7 Hz, 1F, CF) ppm. **^{77}Se NMR** (115 MHz, CD_2Cl_2, -30 °C): δ = 961.42 (m, br)[†] ppm.

[†] Poorly resolved multiplicity of dp (1146, 162 Hz) can be suspected.

Synthesis and characterization of [IPr_2-TeF_3][TeF_5] (3Te) and [*a*IPr-TeF_4] (4Te)

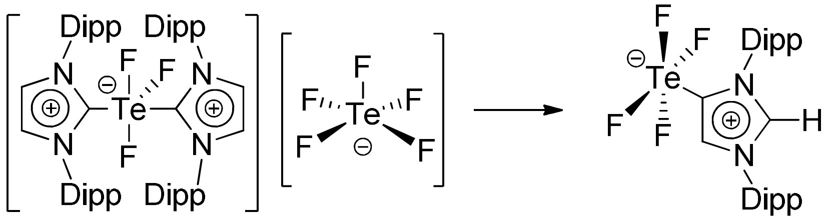

IPr (25 mg, 0.064 mmol) and TeF_4 (13 mg, 0.064 mmol) were combined in a J. Young NMR tube and dissolved in THF-d_8 (0.5 mL). The fresh sample was analysed by NMR and the ongoing reaction was followed over the course of several days (see **Figures S12–S15**). In the initial state [IPr_2TeF_3][TeF_5] (**3Te**) was identified as the main species. As shown in **Table S1** a slow conversion into the abnormal *a*IPrTeF$_4$ complex **4Te** proceeded, reaching 99% conversion after 6 d. At the end of the reaction alongside **6Te** (≈80%) about 8% of [IPrH][TeF_5] and 12% of **IPr** remained. Crystals of **3Te** and **4Te** suitable for SC-XRD were obtained by slow diffusion of hexane into the THF-d_8 solution. Attempts to isolate clean products in macroscopic quantities were unsuccessful. By heating to 40 °C the reaction was completed in 3 d instead of 6 d. Higher temperatures (60–80 °C) caused irreproducibility and sometimes led to different unidentified products, e.g., the small unidentified singlet resonance found at −61 ppm in **Figure S14** became a main product. To be noted is that the oxidation of **1Te** with 1 or 2 eq. of XeF_2 in THF-d_8 gave a very similar mixture to the addition between **IPr** and TeF_4. The ratios of the different ^{19}F NMR signals deviated slightly and some additional unidentified peaks were present. Thus, there was no incentive to pursue the oxidative method with XeF_2 over the TeF_4-based addition.

3Te: ^{1}H NMR (600 MHz, THF-d_8): δ = 7.77 (s, 4H), 7.51 (t, J = 7.8 Hz, 4H), 7.22 (d, J = 7.8 Hz, 8H), 2.33 (hept, J = 6.5 Hz, 8H), 1.04 (d, J = 6.9 Hz, 24H), 0.93 (d, J = 6.8 Hz, 24H) ppm. **^{13}C NMR** (151 MHz, THF-d_8): δ = 146.96, 146.50, 133.19, 132.33, 128.11, 125.19, 29.91, 26.01, 22.98 ppm. **^{19}F NMR** (565 MHz, THF-d_8): δ = −24.12 (t, $^{2}J_{F–F}$ = 22 Hz, $^{1}J_{F–Te}$ = 522 Hz, [IPr_2TeF_3]$^+$), −32.12 (p, $^{2}J_{F–F}$ = 50 Hz, $^{1}J_{F–Te}$ = 2968 Hz, [TeF_5]$^-$), −37.61 (d, $^{2}J_{F–F}$ = 50 Hz, $^{1}J_{F–Te}$ = 1459 Hz, [TeF_5]$^-$), −91.52 (d, $^{2}J_{F–F}$ = 22 Hz, $^{1}J_{F–Te}$ = 776 Hz, [IPr_2TeF_3]$^+$) ppm. **^{125}Te NMR** (190 MHz, THF-d_8): δ = 1144.4 (dp, $^{1}J_{Te–F}$ = 2966, 1458 Hz, [TeF_5]$^-$), 1078.9 (dt, $^{1}J_{Te–F}$ = 520, 774 Hz, [IPr_2TeF_3]$^+$) ppm.

4Te: ^{1}H NMR (600 MHz, THF-d_8): δ = 9.18 (d, J = 1.9 Hz, 1H), 8.14 (d, J = 1.9 Hz, 1H), 7.60 (t, J = 7.7 Hz, 1H), 7.45 (d, J = 7.9 Hz, 2H, overlap), 7.45 (t, 1H, overlap), 7.27 (d, J = 7.8 Hz, 1H), 2.73 (hept, J = 6.8 Hz, 2H), 2.54 (hept, J = 6.9 Hz, 2H), 1.35 (d, J = 6.6 Hz, 6H), 1.28 (d, J = 6.8 Hz, 6H), 1.21 (d, J = 6.9 Hz, 6H), 1.08 (d, J = 7.1 Hz, 6H) ppm. **^{13}C NMR** (151 MHz, THF-d_8): δ = 159.05 (p, J = 28, 27), 147.36, 146.64, 139.45, 134.16, 132.63, 131.27, 130.63, 125.48, 124.22, 30.12, 29.89, 26.44, 24.76, 24.47, 23.02 ppm. **^{19}F NMR** (565 MHz, THF-d_8): δ = −46.98 (s, $^{1}J_{F–Te}$ = 239 Hz) ppm. **^{125}Te NMR** (190 MHz, THF-d_8): δ = 1202.2 (p, $^{1}J_{Te–F}$ = 240 Hz) ppm.

Table S1: Molar ratios (derived from the ^{19}F NMR spectra in **Figure S14**) of the major species found over the course of the reaction between **IPr** and TeF_4 at 23°C.

Time / d	$[TeF_5]^-$ (counter ion in 3Te and of $[IPrH]^+$)	**3Te** ($[IPr_2TeF_3]^+$)	**4Te**
0	1	0.82	0.01
1	1	0.58	0.93
6	0.12	0.01	1

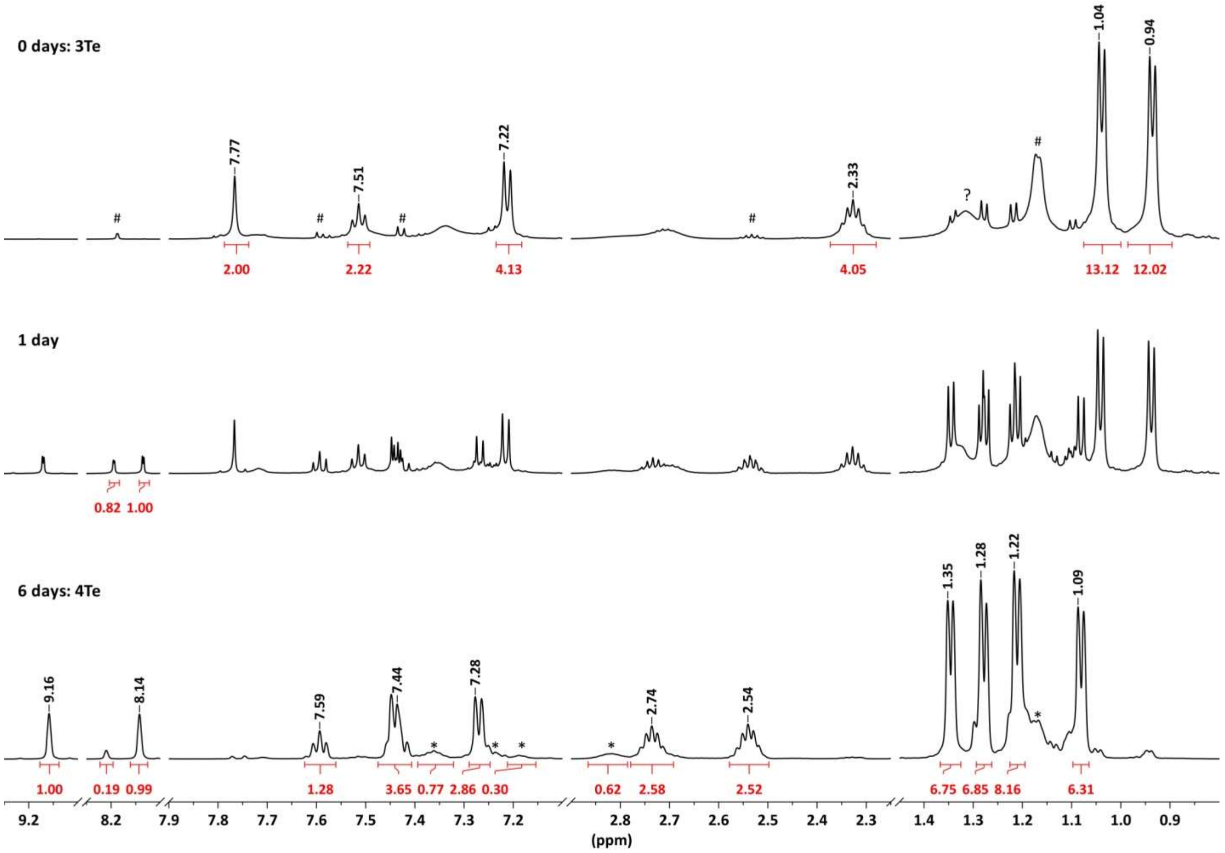

Figure S12: ^1H NMR (600 MHz, THF-d_8) spectra following the reaction between **IPr** and TeF_4. Signals of **3Te** and **4Te** have been marked (* denotes IPr, # denotes $[IPrH]^+$, ? denotes an unknown impurity).

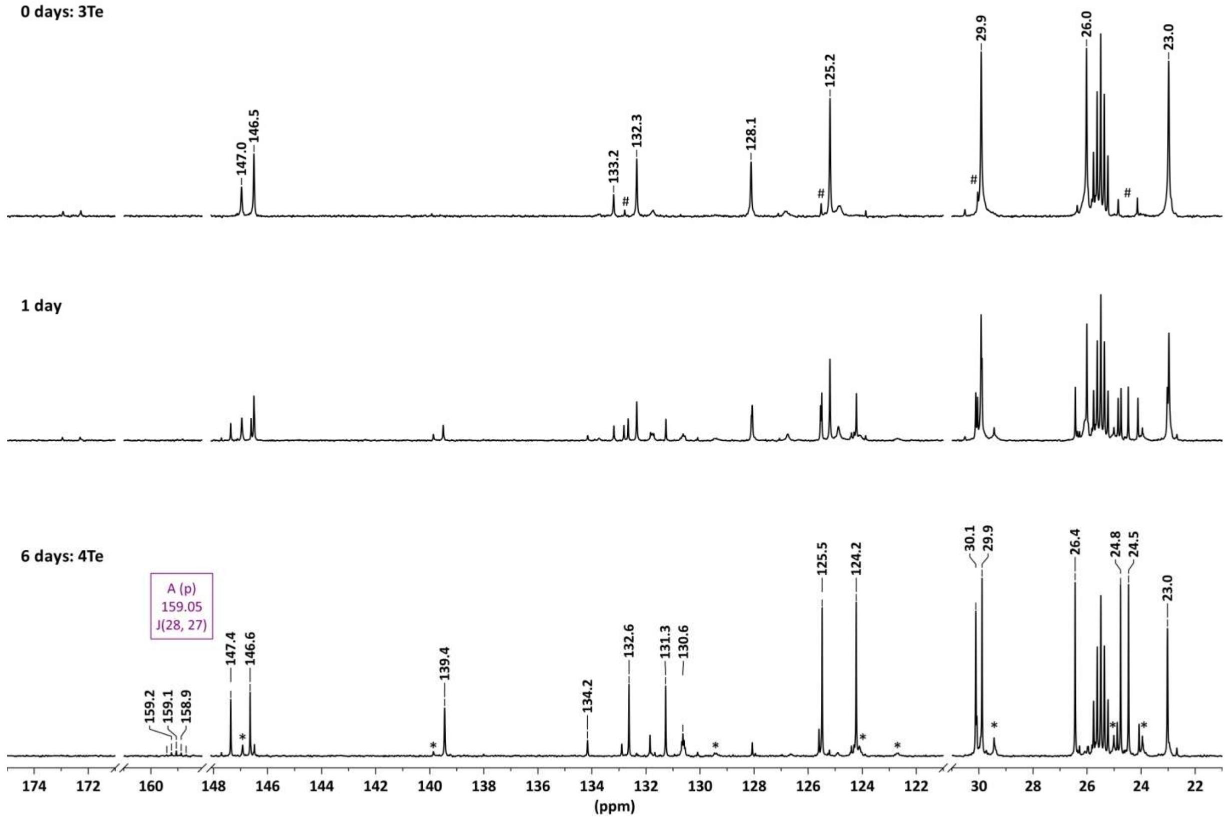

Figure S13: ^{13}C NMR (151 MHz, THF-d_8) spectra following the reaction between **IPr** and TeF_4. Chemical shifts of the signals of **3Te** (top) and **4Te** (bottom) have been marked (* denotes IPr, # denotes $[IPrH]^+$).

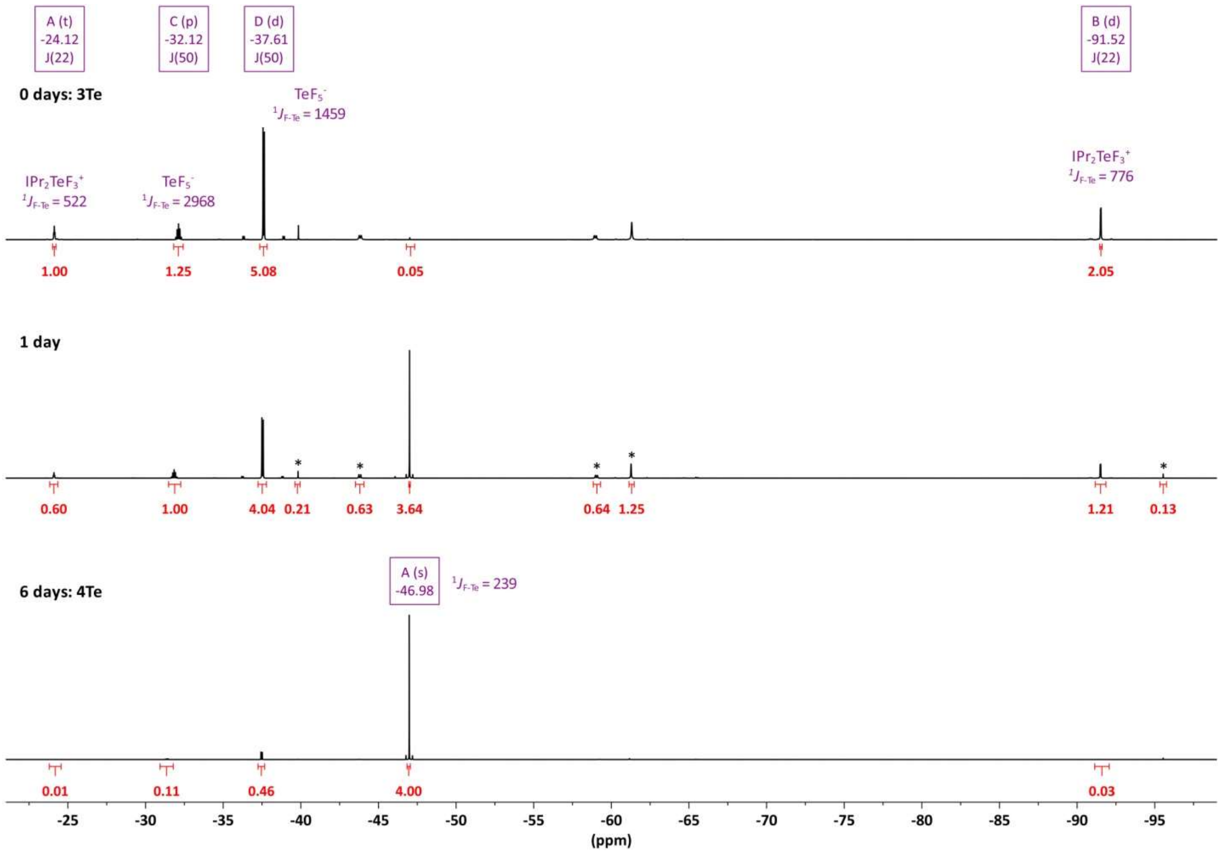

Figure S14: ^{19}F NMR (565 MHz, THF-d_8) spectra following the reaction between **IPr** and TeF_4. Signals of **3Te** (top) and **4Te** (bottom) and $^{1}J(^{125}Te–^{19}F)$ coupling constants have been marked (* denotes unknown impurities).

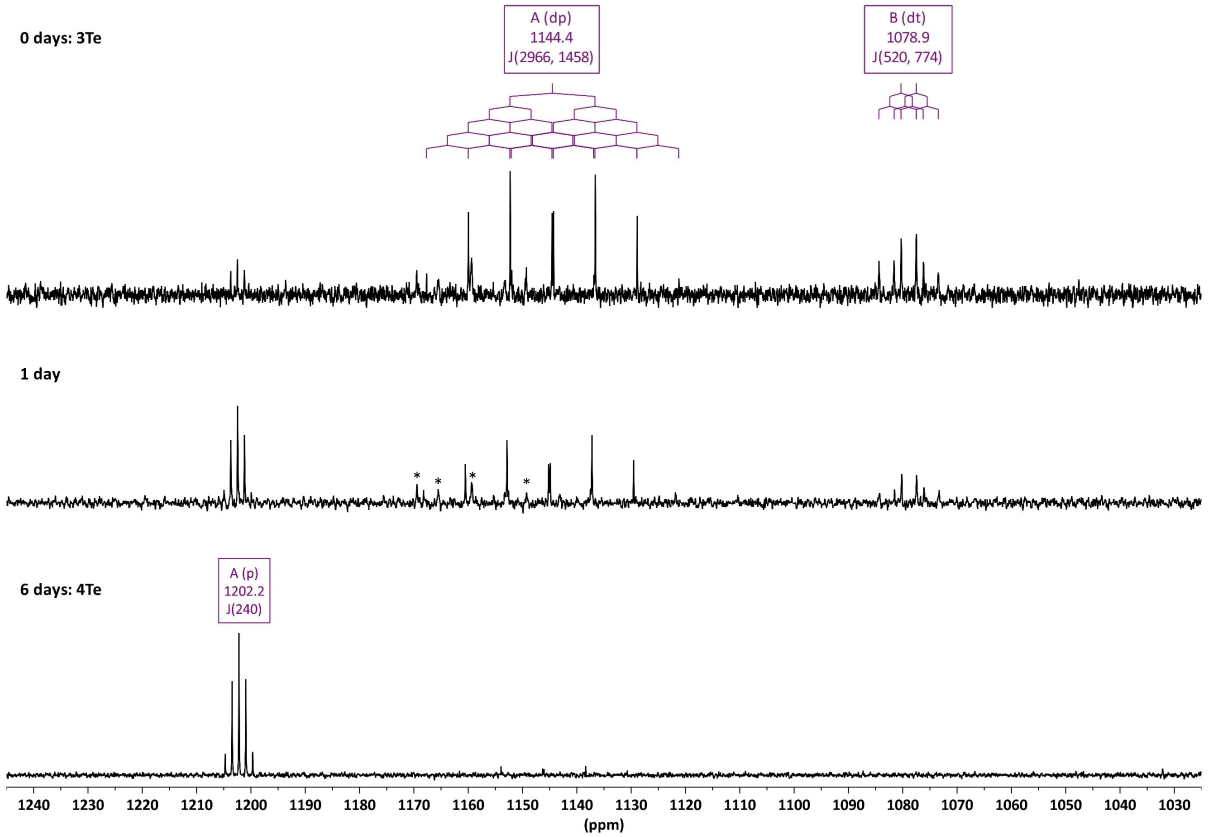

Figure S15: ^{125}Te (190 MHz, THF-d_8) NMR spectra following the reaction between **IPr** and TeF_4. Multipletts of **3Te** (top) and **4Te** (bottom) have been marked (* denotes unidentified signals).

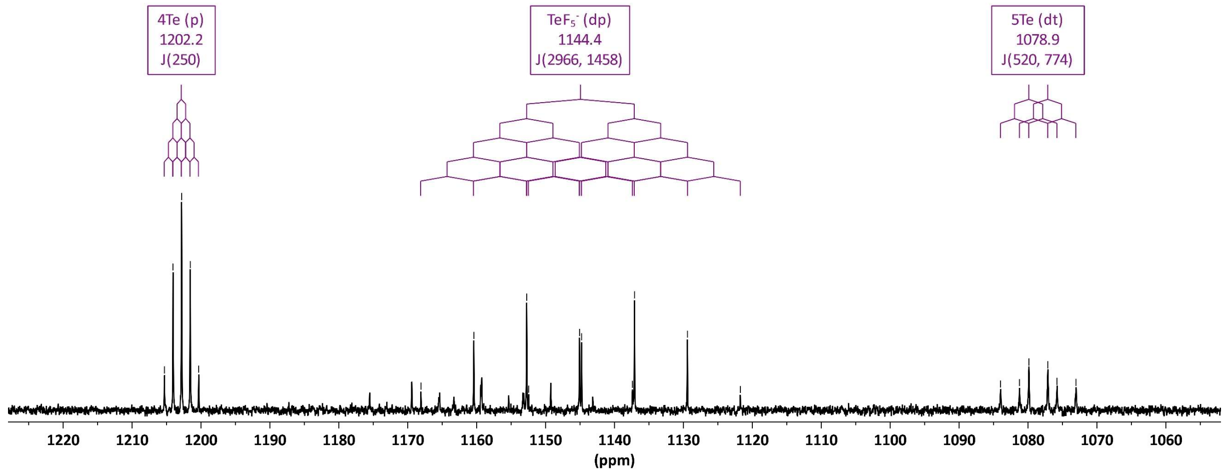

Figure S16: ^{125}Te (190 MHz, THF-d_8) NMR spectrum measured after heating a mixture of **IPr** and TeF4 to 80°C for 2 h in a J. Young NMR tube. Alongside **3Te** (with a [TeF_5] counterion) and **4Te** some unidentified signals are present between 1180 and 1140 ppm.

Attempted Synthesis of (I^{i}Pr$_2$Me$_2$)SF$_2$

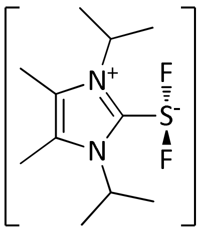

THF (30 mL) was added to a pre-cooled (−78 °C) solid mixture of (I^{i}Pr$_2$Me$_2$)SCl$_2$ (0.570 g, 2.01 mmol) and AgF (0.510 g, 4.02 mmol), according to Kuhn *et al.*[S8] The suspension was left to slowly warm up over 24 h and the supernatant was isolated using a syringe (equipped with a filter). After removal of the solvent *in vacuo* the remaining amorphous orange solid was extracted by adding 5 mL of diethylether and stirring for 3 d. This was carried out instead of the prescribed recrystallisation from Et$_2$O/*n*-Pentane, since no immediate solubility in Et$_2$O was visible. NMR Samples were taken from the crude solid, the Et$_2$O insoluble solid, and the Et$_2$O extract. The major peaks in the ^{1}H NMR spectra of all three phases conform to the signals reported by Kuhn *et al.*[S8] but also match to those of (I^{i}PrMe)S. The reported ^{19}F NMR signal (37.7 ppm)[S8] was found as a trace in the crude mixture and the Et$_2$O extract.

Signals reported by Kuhn *et al.*: **^{1}H-NMR** (60 MHz, C$_6$D$_6$, TMS int.): δ = 5.89 (br s, 2H), 1.59 (s, 6H), 1.15 (br s, 12H) ppm. **^{19}F-NMR** (75.39 MHz, C$_6$D$_6$, CFCl$_3$, ext.): δ = 37.7 ppm.[S10]

Found signals (Et$_2$O extract): **^{1}H NMR** (600 MHz, C$_6$D$_6$): δ = 6.04 (s, br, 2H), 1.58 (s, 6H), 1.17 (s, br, 12H) ppm. **^{19}F NMR** (565 MHz, C$_6$D$_6$): δ = −37.86, −39.04, −82.41 ppm. Crude mixture: **^{19}F NMR** (565 MHz, C$_6$D$_6$): δ = 45.07, 37.83, −105.43 ppm. **^{13}C NMR** (151 MHz, C$_6$D$_6$): δ = 120.77, 67.83, 49.25, 25.80, 22.10, 20.63, 9.88, 1.42.

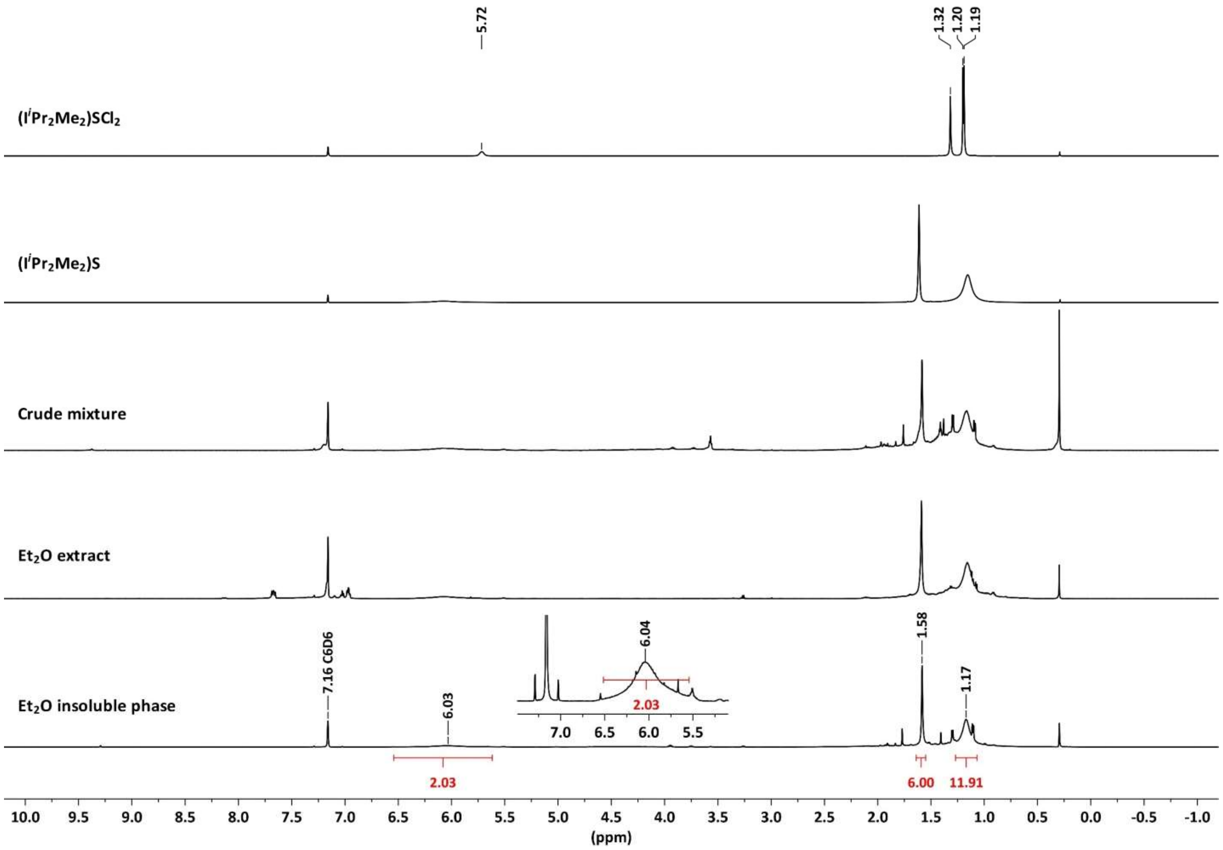

Figure S17: ^{1}H NMR (600 MHz, C_6D_6) spectra of (I^{i}Pr$_2$Me$_2$)SCl$_2$, (I^{i}Pr$_2$Me$_2$)S and the three phases obtained in the reaction between (I^{i}Pr$_2$Me$_2$)SCl$_2$ and AgF.

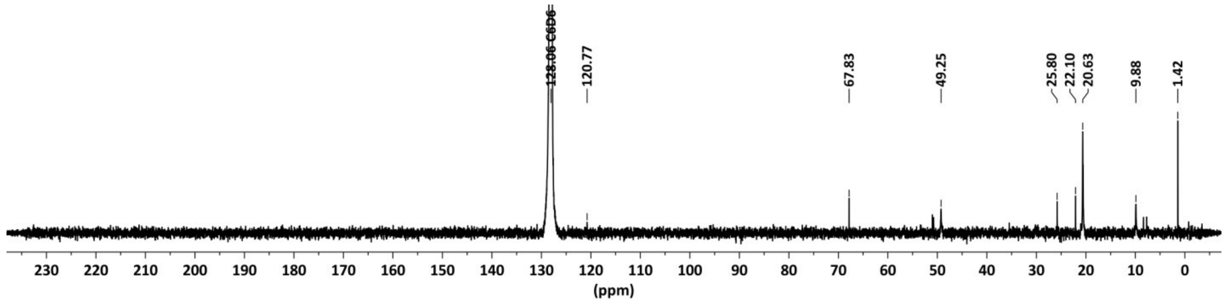

Figure S18: ^{13}C NMR (151 MHz, C_6D_6) spectrum of the crude reaction mixture between $(I^iPr_2Me_2)SCl_2$ and AgF.

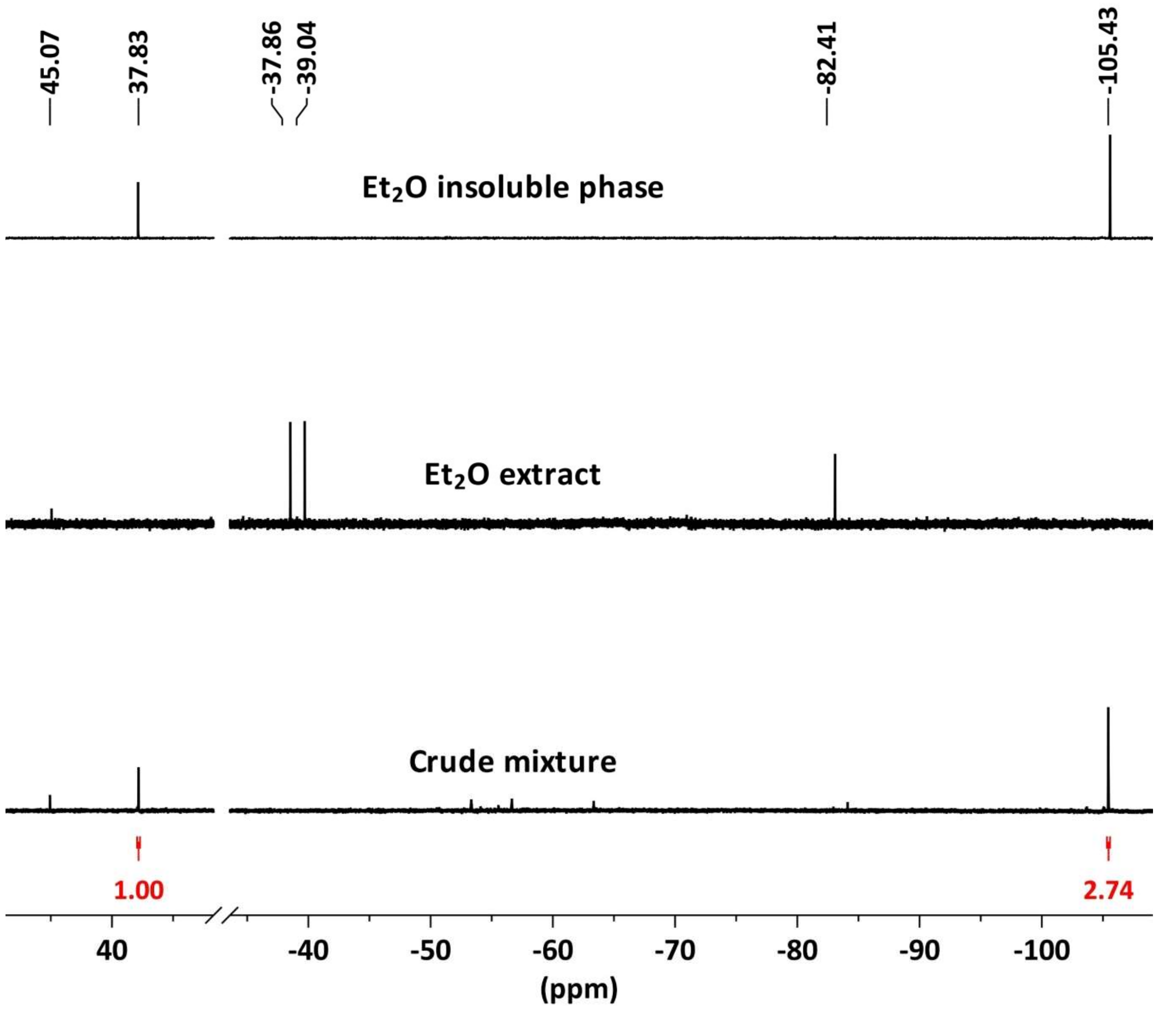

Figure S19: ^{19}F NMR (565 MHz, C_6D_6) spectra of the three different phases obtained from the reaction between (I^{i}Pr$_2$Me$_2$)SCl$_2$ and AgF.

Crystallographic data

Intensity data of **2S**, **2Se**·1.5 $C_6H_2F_2$, **3Se**, **4Se**·THF, **3Te** and **4Te**·THF was collected on a Bruker Venture D8 diffractometer at 100 K with graphite-monochromatic Mo-Kα (0.7107 Å) radiation. All structures were solved by direct methods and refined based on F2 by use of the SHELX program package as implemented in WinGX[S11,S12] or OLEX2.[S13] All non-hydrogen atoms were refined using anisotropic displacement parameters. Hydrogen atoms attached to carbon atoms were included in geometrically calculated positions using a riding model. Crystal and refinement data are collected in **Tables S2** and **S3**. Figures were created using DIAMOND.[S14] Crystallographic data for the structural analyses have been deposited with the Cambridge Crystallographic Data Centre. Copies of this information may be obtained free of charge from The Director, CCDC, 12 Union Road, Cambridge CB2 1EZ, UK (Fax: +44-1223-336033; e-mail: deposit@ccdc.cam.ac.uk or http://www.ccdc.cam.ac.uk).

Table S2. Crystal data and details of the structure determination for **2S**, **2Se·1.5 $C_6H_2F_2$**, **3Se** and **4Se**·THF.

	**2S**	**2Se**·1.5 $C_6H_2F_2$	**3Se**	**4Se**·THF
**CCDC number**	2143921	2143922	2143923	2143924
**Empirical formula**	$C_{27}H_{36}F_2N_2S$	$C_{36}H_{42}F_5N_2Se$	$C_{27}H_{36}F_4N_2Se$	$C_{31}H_{44}F_6N_2OSe$
**Formula weight**	458.64	676.67	543.54	653.64
**Temperature/K**	100.01	99.94	99.99	100.01
**Crystal system**	monoclinic	monoclinic	orthorhombic	monoclinic
**Space group**	$P2_1/c$	$P2_1/c$	Pnma	$P2_1/c$
**a/Å**	20.4347(18)	14.0030(8)	11.9571(5)	10.8106(9)
**b/Å**	19.207(2)	19.3478(14)	19.8975(9)	25.6200(18)
**c/Å**	13.8964(17)	25.4169(18)	10.8443(5)	12.2502(10)
**α/°**	90	90	90	90
**β/°**	109.560(4)	99.316(3)	90	108.753(3)
**γ/°**	90	90	90	90
**Volume/Å^3**	5139.5(9)	6795.3(8)	2580.0(2)	3212.8(4)
**Z**	8	8	4	4
**ρ_{calc}g/cm^3**	1.185	1.323	1.399	1.351
**μ/mm^{-1}**	0.157	1.159	1.502	1.229
**F(000)**	1968.0	2808.0	1128.0	1360.0
**Crystal size/mm^3**	0.5 × 0.4 × 0.4	0.3 × 0.3 × 0.3	0.3 × 0.3 × 0.3	0.4 × 0.4 × 0.4
**Radiation**	MoKα (λ = 0.71073)	MoKα (λ = 0.71073)	MoKα (λ = 0.71073)	MoKα (λ = 0.71073)
**2Θ range for data collection/°**	4.23 to 56.566	3.77 to 56.564	4.278 to 61.016	3.854 to 62.032
**Index ranges**	$-23 \le h \le 27$<br>$-25 \le k \le 25$<br>$-18 \le l \le 18$	$-16 \le h \le 18$<br>$-25 \le k \le 25$<br>$-33 \le l \le 33$	$-17 \le h \le 17$<br>$-28 \le k \le 28$<br>$-15 \le l \le 15$	$-15 \le h \le 15$<br>$-37 \le k \le 37$<br>$-17 \le l \le 17$
**Reflections collected**	162769	217855	74258	82530
**Independent reflections**	12777<br>R_{int} = 0.0635<br>R_{sigma} = 0.0283	16864<br>R_{int} = 0.0457<br>R_{sigma} = 0.0195	4046<br>R_{int} = 0.0398<br>R_{sigma} = 0.0152	10252<br>R_{int} = 0.0497<br>R_{sigma} = 0.0296
**Data/restraints/parameters**	12777/0/593	16864/13/881	4046/0/161	10252/0/378
**Goodness-of-fit on F^2**	1.019	1.018	1.074	1.054
**Final R indexes [I>=2σ (I)]**	R_1 = 0.0488<br>wR_2 = 0.1237	R_1 = 0.0288<br>wR_2 = 0.0667	R_1 = 0.0244<br>wR_2 = 0.0643	R_1 = 0.0364<br>wR_2 = 0.0856
**Final R indexes [all data]**	R_1 = 0.0633<br>wR_2 = 0.1331	R_1 = 0.0395<br>wR_2 = 0.0723	R_1 = 0.0292<br>wR_2 = 0.0669	R_1 = 0.0510<br>wR_2 = 0.0914
**Largest diff. peak/hole/eÅ^{-3}**	0.69/-0.72	0.43/-0.39	0.40/-0.50	0.86/-0.59

Table S3. Crystal data and details of the structure determination for**3Te** and **4Te**·THF.

	**3Te**	**4Te**·THF
**CCDC number**	2143925	2143926
**Empirical formula**	$C_{13.5}H_{18}F_2NTe_{0.5}$	$C_{31}H_{44}F_4N_2OTe$
**Formula weight**	296.09	664.28
**Temperature/K**	170.00	100.00
**Crystal system**	triclinic	monoclinic
**Space group**	$P\bar{1}$	$P2_1/n$
**a/Å**	9.9095(4)	11.2246(5)
**b/Å**	12.4780(5)	16.0211(8)
**c/Å**	12.6363(5)	17.7349(8)
**α/°**	71.657(2)	90
**β/°**	74.203(2)	105.355(2)
**γ/°**	68.450(2)	90
**Volume/Å^3**	1357.54(10)	3075.4(2)
**Z**	4	4
**ρ_{calc}g/cm^3**	1.449	1.435
**μ/mm^{-1}**	1.140	1.017
**F(000)**	600.0	1360.0
**Crystal size/mm^3**	0.15 × 0.15 × 0.1	0.4 × 0.25 × 0.1
**Radiation**	MoKα (λ = 0.71073)	MoKα (λ = 0.71073)
**2Θ range for data collection/°**	4.49 to 66.564	4.542 to 59.15
**Index ranges**	-15 ≤ h ≤ 15<br>-19 ≤ k ≤ 19<br>-19 ≤ l ≤ 19	-15 ≤ h ≤ 15,<br>-22 ≤ k ≤ 22,<br>-24 ≤ l ≤ 24
**Reflections collected**	108764	129145
**Independent reflections**	10463<br>R_{int} = 0.0419<br>R_{sigma} = 0.0234	8641<br>R_{int} = 0.0458<br>R_{sigma} = 0.0169
**Data/restraints/parameters**	10463/0/360	8641/0/369
**Goodness-of-fit on F^2**	0.928	1.076
**Final R indexes [I>=2σ (I)]**	R_1 = 0.0337<br>wR_2 = 0.1096	R_1 = 0.0210<br>wR_2 = 0.0523
**Final R indexes [all data]**	R_1 = 0.0456<br>wR_2 = 0.1194	R_1 = 0.0244<br>wR_2 = 0.0538
**Largest diff. peak/hole/eÅ^{-3}**	0.53/-0.49	1.28/-0.52

Computational details.

The bonding situation between the carbene (IPr) and the EF_2 or EF_4 fragments (E = S, Se, Te) was comprehensively studied by means of density functional theory (DFT) computations. The bond dissociation energies (E_d) connecting the molecular fragments were estimated by a variety of approaches including potential energy scans (PES) of the complexes, energy decomposition analysis and unrestrained optimization. In addition, a set of topological, surface, and integrated real-space bonding indicators was determined for all complexes from the calculated electron and electron pair densities applying the Atoms-In-Molecules, noncovalent interactions index, and electron localizability indicator approaches to receive bond paths motifs, contact patches, atomic and fragmental charges, and electron populations in bonding and lone-pair (LP) basins. Starting from the available solid-state molecular geometries density functional theory (DFT) computations were performed in the gas-phase at the B3PW91/6-311+G(2df,p)[S15] level of theory using Gaussian09.[S16] For the Te atoms, effective core potentials (ECP28MDF)[S17] and corresponding cc-pVTZ basis set[S17] were utilized. Dispersion was taken account for by the empirical disperison correction of Grimme.[S18] Energy decompositon analysis (EDA)[S19] was performed with the Amsterdam Density Functional (ADF)[S20] software at the B3PW91/TZ2P level of theory[S21] using the "core none" option and including Grimme's D4 dispersion correction.[S18] The wavefunction files were used for a topological analysis of the electron density according to the Atoms-In-Molecules space-partitioning scheme[S22] using AIM2000,[S23] whereas DGRID[S24] was used to generate and analyze the Electron-Localizability-Indicator (ELI-D)[S25,S26] related real-space bonding descriptors applying a grid step size of 0.05 a.u. (0.12 a.u. for visualization). The NCI[S27] grids were computed with NCIplot (0.1 a.u. grids).[S28] Bond paths are displayed with AIM2000, ELI-D and NCI figures are displayed with MolIso,[S29] and spin densities are displayed with GaussView. AIM provides a bond paths motif, which resembles and exceeds the Lewis picture of chemical bonding, disclosing all types and strengths of interactions. Additionally, it provides atomic volumes and charges. Analyses of the reduced density gradient, $s(\mathbf{r}) = [1/2(3\pi^2)^{1/3}]|\nabla\rho|/\rho^{4/3}$, according to the NCI method is used to visualize non-covalent bonding aspects. An estimation of different non-covalent contact types according to steric/repulsive ($\lambda_2 > 0$), van der Waals-like ($\lambda_2 \approx 0$), and attractive ($\lambda_2 < 0$) is facilitated by mapping the ED times the sign of the second eigenvalue of the Hessian ($\text{sign}(\lambda_2)\rho$) on the *iso*-surfaces of $s(\mathbf{r})$. AIM and NCI are complemented by the ELI-D, which provides electron populations and volumes of bonding and lone-pair basins and is especially suitable for the analysis of (polar-) covalent bonding aspects.

The optimized free EF_2 molecules are angled (F–E–F ≈ 94-98°) and the EF_4 molecules form a trigonal bipyramid with two axial and two equatorial F atoms and the LP of S, Se, or Te in equatorial position, see Figure S21. In the donor-acceptor adducts, however, the EF_2 or EF_4 parts are considerably flattened with all F–E–F angles larger than 165° (opposing F atoms in EF_4), see Figure 4 (article) and Figure S22 and S23–S28. Accordingly, molecular reorganization

can not be neglected in a bonding analysis. In order to reliably deconvolute bond dissociation from molecular reorganization in $IPrEF_2$ and $IPrEF_4$, four different computational approaches were pursued and compared (see Figure S21 and Tables S4 and S5). The focus was laid on S and Se containing compounds; all computations were conducted with and without empirical dispersion correction.

Source 1: The relaxed gas-phase geometries of $IPrEF_2$ and $IPrEF_4$ were used for potential energy scans (PES) by systematically varying the E–C distances up to 7 or 7.15 Å, see Figure S20. For each fixed E–C distance a single-point calculation as well as a restrained optimization was performed, resulting in typical trend lines, which allow to discriminate bond dissociation from molecular reorganization. The difference between the energy values at the maximal distances and zero are listed in Table S4, and are denoted as E_{sp} (single-point), E_{opt} (optimization), and E_{diff} (E_{sp}-E_{opt}).

Source 2: The EF_2 and EF_4 parts of the optimized complexes were extracted and used as starting geometries for optimizations, giving reorganization energies (E_{re}), see Table S4. Due to minor structural variation, molecular reorganization of the IPr fragment was neglected.
Source 3: The fragments IPr, EF_2 and EF_4 were individually optimized in the gas-phase; the sum of IPr and EF_2 or EF_4 was then compared to the molecular energy of the corresponding $IPrEF_2$ or $IPrEF_4$ complexes (A + B vs. AB), giving fragment sum difference energies (E_{fs}), see Table S4.
Source 4: Energy decomposition analysis (EDA) was conducted for $IPrEF_2$ and $IPrEF_4$ (including Te) to determine the respective contributions of Pauli repulsion (E_{Pauli}), electrostatic attraction (E_{elstat}), orbital attraction (E_{orb}), and eventually dispersion (E_{disp}) to the total (or instantaneous) interaction energy (E_{int}) between the IPr and the EF_2 or EF_4 fragments, see Table S5. E_{int} is also listed in Table S4. Close inspection of Table S4 reveals three pairs of energies, the two energies of each pair basically containing the same information. 1. The total interaction energy obtained from EDA (E_{int}) is similar to the single-point energy in PES at large distance (E_{sp}), which is expected. Differences may stem from the different levels of DFT and remains of weak secondary interactions between the IPr and the EF_2 or EF_4 fragments in PES, even at 7 or 7.15 Å. 2. The fragment sum energy (E_{fs}) obtained from separate optimization of fragments and complexes is similar to the energy for restrained (fixed E–C distance) optimizations in PES at large distances (E_{opt}). These energies are estimates for the bond dissociation energy (E_d) connecting the carbene with the chalcogen halides. Differences are small for EF_2, supporting the idea that relaxation of IPr is negligible, but large for EF_4, which may be caused by incomplete relaxation of the EF_4 fragments even at 7 or 7.15 Å in PES. The reorganization energy of the EF_2 or EF_4 fragments (E_{re}) are similar to the difference of E_{sp} and E_{opt} in PES (E_{diff}). The fact that $E_{re}(EF_4)$ is as strong as $E_{re}(EF_2)$ in the gas-phase supports the assumption that the relaxation of EF_4 is not complete even at 7 or 7.15 Å in PES. Disregarding these limitations, the

parallel and independent estimation of the total interaction energy, the bond dissociation energy and the molecular reorganization energy provides confidence in the reliability of the numbers. Dispersion effects (ΔEF_2 or ΔEF_4) range from 50–70 kJ mol^{-1} and are smaller for S than for Se containing compounds, and smaller for EF_2 than for EF_4 containing compounds, as expected.

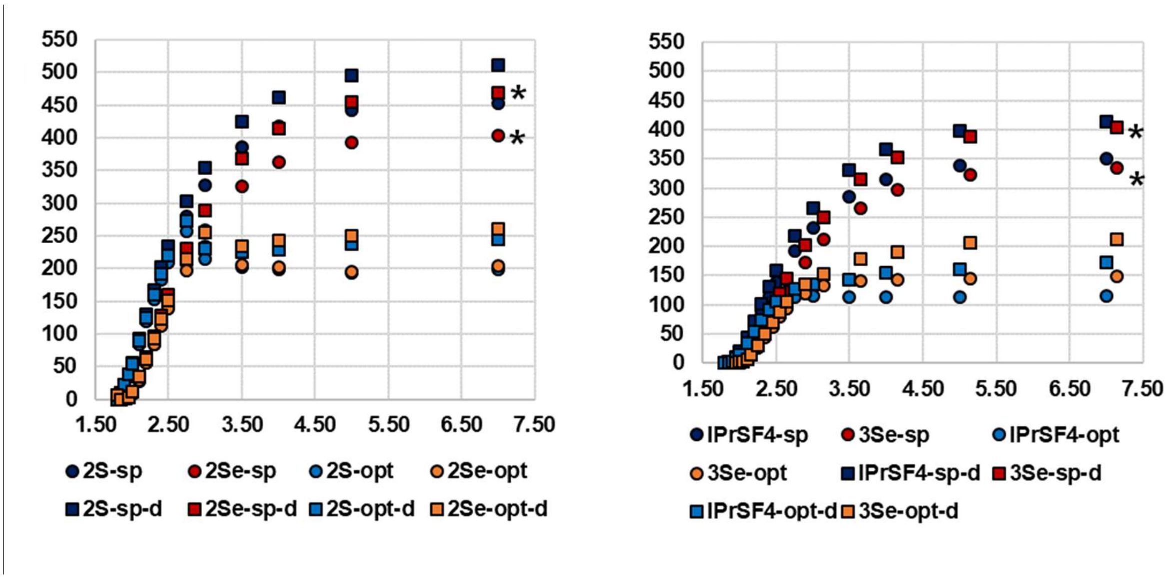

Figure S20 Potential energy scans for (left) **2S**, **2Se** and (right) **IPrSF₄** and **3Se**. **2S** and **IPrSF₄** are colored dark or light blue, **2Se** and **3Se** red or orange. Single-point calculations are colored dark blue or red, geometry optimizations light blue or orange. Calculations without dispersion correction are denoted by circles, with dispersion by squares. The lowest energies were set to zero. All values are in kJ/mol. Notably, single point calculations of **2Se** and **3Se** at Se–C distances of 7.0 or 7.15 Å did not lead to stable solutions, with or without including dispersion correction (red circles and squares, highlighted by *). These numbers were estimated by keeping fixed differences to the corresponding numbers of **2S** and **IPrSF₄**, which were obtained at a S–C distance of 5.0 Å (blue circle and squares), resulting in meaningful progressions of the observed trend lines.

Table S4 Energy discriminators (in kJ mol^{-1})

	Source 1: PES							Source 2	
	single-point E_{sp}		optimization E_{opt}		difference E_{diff}			reorganization E_{re}	
	S	Se	S	Se	S	Se		S	Se
IPrEF$_2$	–453	–404	–198	–204	–255	–200	EF$_2$	–267	–211
IPrEF$_4$	–350	–334	–116	–148	–235	–186	EF$_4$	–265	–201
IPrEF$_2$(d)	–511	–470	–245	–261	–266	–209	EF$_2$(d)	–266	–210
IPrEF$_4$(d)	–414	–403	–173	–211	–241	–192	EF$_4$(d)	–266	–202
ΔIPrEF$_2$	–58	–66	–47	–57	–11	–9	ΔEF$_2$	0	0
ΔIPrEF$_4$	–64	–69	–57	–63	–6	–5	ΔEF$_4$	–1	–1

	Source 3 fragment sum E_{fs}		Source 4 EDA E_{int}	
	S	Se	S	Se
IPrEF$_2$	–197	–198	–466	–411
IPrEF$_4$	–69	–115	–353	–329
IPrEF$_2$(d)	–246	–255	–521	–473
IPrEF$_4$(d)	–126	–179	–414	–395
ΔIPrEF$_2$	–49	–57	–56	–62
ΔIPrEF$_4$	–58	–64	–61	–66

	no dispersion					
	E_{sp}-E_{EDA}		E_{opt}-E_{fs}		E_{diff}-E_{re}	
	S	Se	S	Se	S	Se
IPrEF$_2$	13	7	–2	–6	12	11
IPrEF$_4$	2	–6	–47	–33	30	15

	dispersion					
	E_{sp}-E_{EDA}		E_{opt}-E_{fs}		E_{diff}-E_{re}	
	S	Se	S	Se	S	Se
IPrEF$_2$	11	3	1	–6	0	1
IPrEF$_4$	0	–8	–47	–32	25	10

All Energies are difference energies, see main text for details. IPr/EF$_2$(d) and EF$_4$(d) refer to calculations including dispersion correction. ΔEF$_2$ and ΔEF$_4$ are the difference between calculations without or with dispersion correction (thus might be denoted as E_{disp}).

Table S5 Energy decomposition analysis (EDA; values are in atomic units or percent)

	E_{int}	E_{Pauli}	E_{elstat}	% E_{elstat}	E_{orb}	% E_{orb}	E_{disp}	% E_{disp}
**2S**	–465.600	1660.230	-987.510	46	–1138.320	54		
IPrSF_4	–352.700	1525.170	-906.810	48	–971.060	52		
**2Se**	–410.680	1335.730	-908.200	52	–838.220	48		
**3Se**	–328.810	1230.580	-821.790	53	–737.600	47		
IPrTeF_2	–344.940	985.070	-765.340	58	–564.670	42		
IPrTeF_4	–296.540	898.700	-691.470	58	–503.760	42		
**2S_disp**	–521.400	1698.710	-1004.630	45	–1155.020	52	–60.460	3
**IPrSF_4_disp**	–413.790	1563.670	-922.700	47	–986.560	50	–68.210	3
**2Se_disp**	–472.640	1382.260	-931.800	50	–856.440	46	–66.660	4
**3Se_disp**	–394.660	1274.750	-842.600	50	–752.960	45	–73.840	4
**IPrTeF_2_disp**	–413.410	1044.470	-799.210	55	–583.870	40	–74.800	5
**IPrTeF_4_disp**	–367.660	950.140	-718.950	55	–519.470	39	–79.370	6

Table **S6** Reorganization energies (E_{re}) and fragment sums (Efs)

Source 2	start (a.u.)		end (a.u.)		Δ (a.u.)		Δ (kJ/mol)	
E_{re}	gas	disp	gas	disp	gas	disp	gas	disp
SF_2	–597.697	–597.699	–597.799	–597.800	–0.102	–0.101	–267	–266
SeF_2	–2601.095	–2601.097	–2601.175	–2601.177	–0.080	–0.080	–211	–210
TeF_2	–467.770	–467.773	–467.828	–467.830	–0.058	–0.057	–152	–151
SF_4	–797.381	–797.385	–797.482	–797.487	–0.101	–0.101	–265	–266
SeF_4	–2800.789	–2800.794	–2800.866	–2800.870	–0.077	–0.077	–201	–202
TeF_4	–667.499	–667.504	–667.553	–667.558	–0.054	–0.055	–142	–143
**IPr**			–1159.938	–1160.093				

Source 3	adducts (a.u.)		NHC + end (a.u.)		Δ (a.u.)		Δ (kJ/mol)	
E_{fs}	gas	disp	gas	disp	gas	disp	gas	disp
**2S**	–1757.812	–1757.987	–1757.737	–1757.893	–0.075	–0.094	–197	–246
**2Se**	–3761.189	–3761.367	–3761.113	–3761.270	–0.075	–0.097	–198	–255
IPrTeF_2	–1627.836	–1628.019	–1627.766	–1627.923	–0.070	–0.096	–184	–253
IPrSF_4	–1957.446	–1957.627	–1957.420	–1957.579	–0.026	–0.048	–69	–126
**3Se**	–3960.848	–3961.031	–3960.804	–3960.963	–0.044	–0.068	–115	–179
IPrTeF_4	–1827.543	–1827.731	–1827.491	–1827.651	–0.052	–0.080	–138	–211

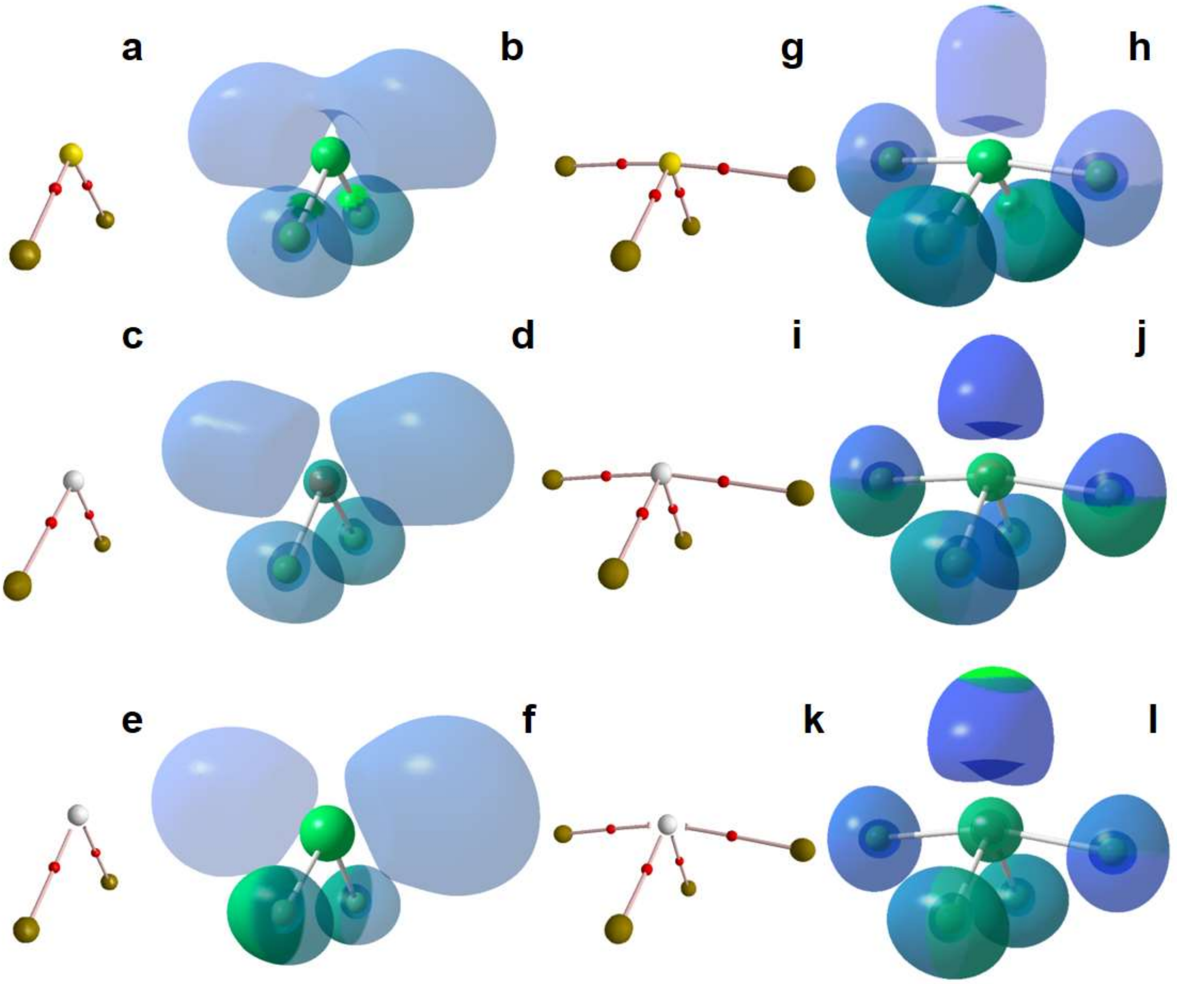

Figure S21 RSBI analysis of SF_2, SeF_2, TeF_2, SF_4, SeF_4, and TeF_4. (a, c, e, g, i, k) AIM bond paths motifs, (b, d, f, h, j, l) ELI-D localization domain representation at *iso*-value of 1.3.

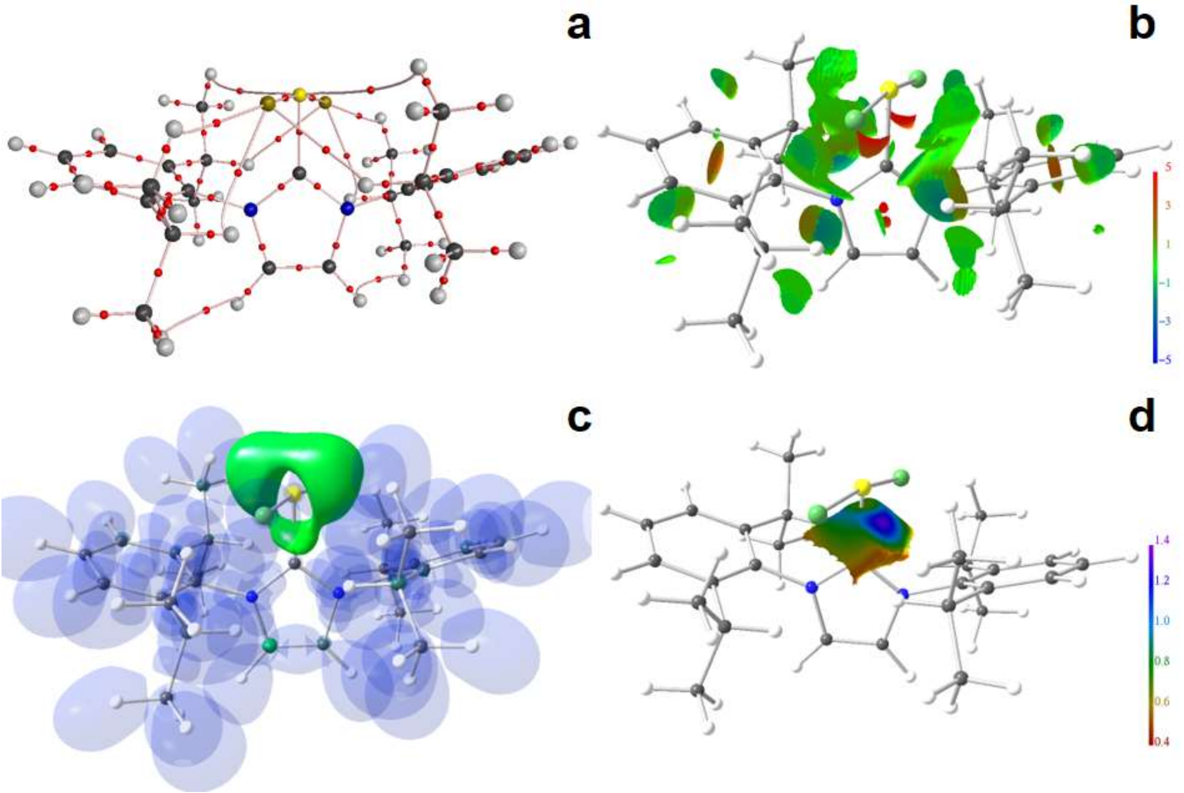

Figure S22 RSBI analysis of **2S** (a) AIM bond paths motif, (b) NCI *iso*-surface at s(**r**) = 0.5, (c) ELI-D localization domain representation at *iso*-value of 1.3, (d) ELI-D distribution mapped on the S–C ELI-D bonding basin.

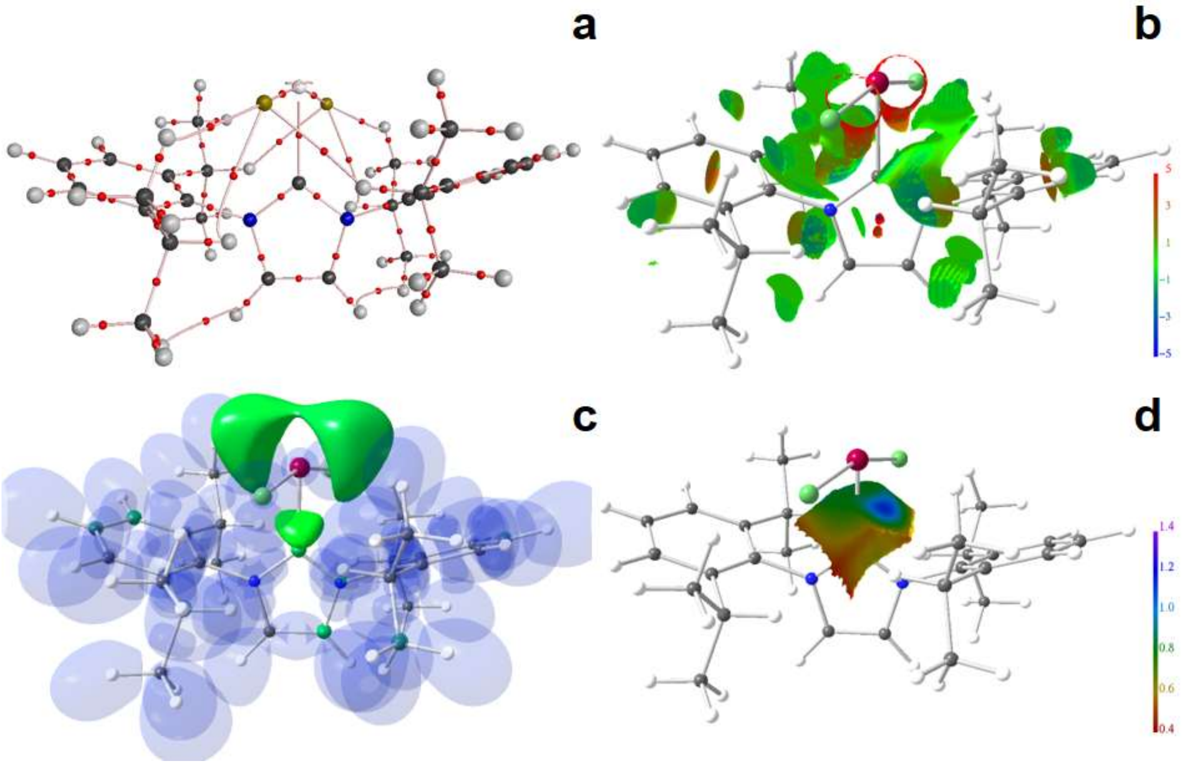

Figure S23 RSBI analysis of experimentally unobserved $IPrTeF_2$ (a) AIM bond paths motif, (b) NCI *iso*-surface at s(**r**) = 0.5, (c) ELI-D localization domain representation at *iso*-value of 1.3, (d) ELI-D distribution mapped on the Te–C ELI-D bonding basin.

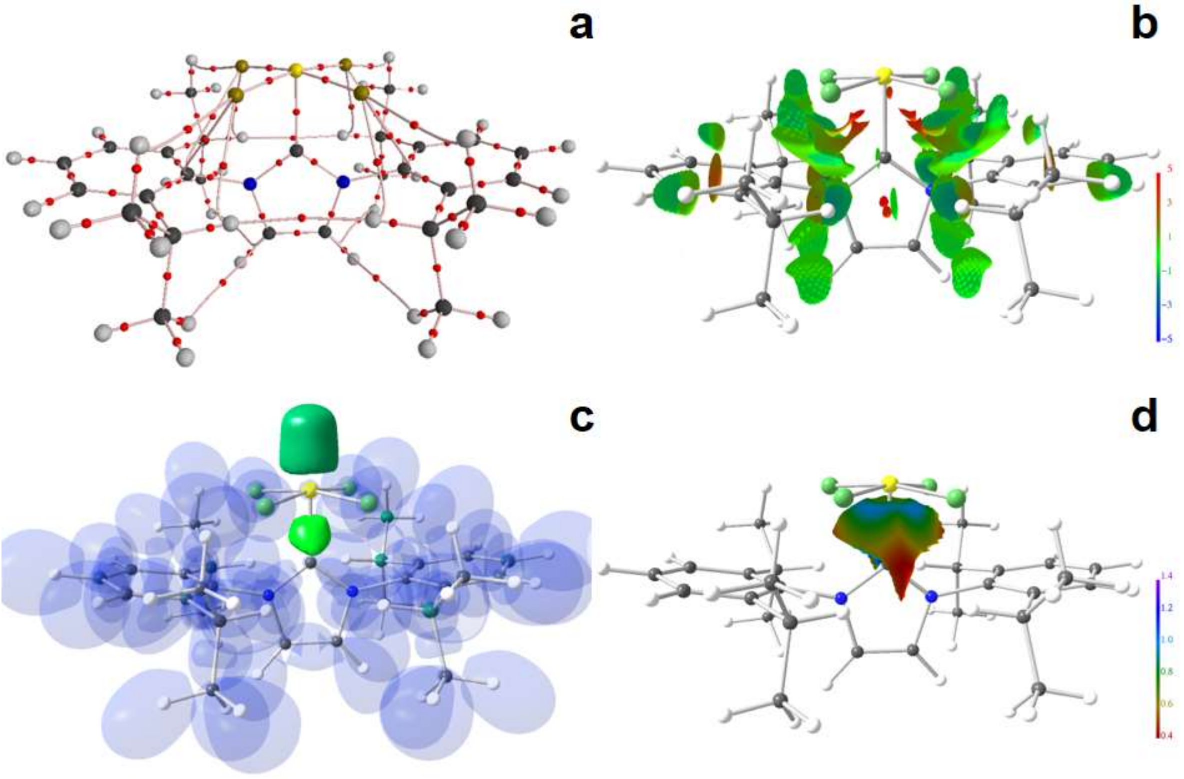

Figure S24 RSBI analysis of experimentally unobserved $IPrSF_4$ (a) AIM bond paths motif, (b) NCI *iso*-surface at s(**r**) = 0.5, (c) ELI-D localization domain representation at *iso*-value of 1.3, (d) ELI-D distribution mapped on the S–C ELI-D bonding basin.

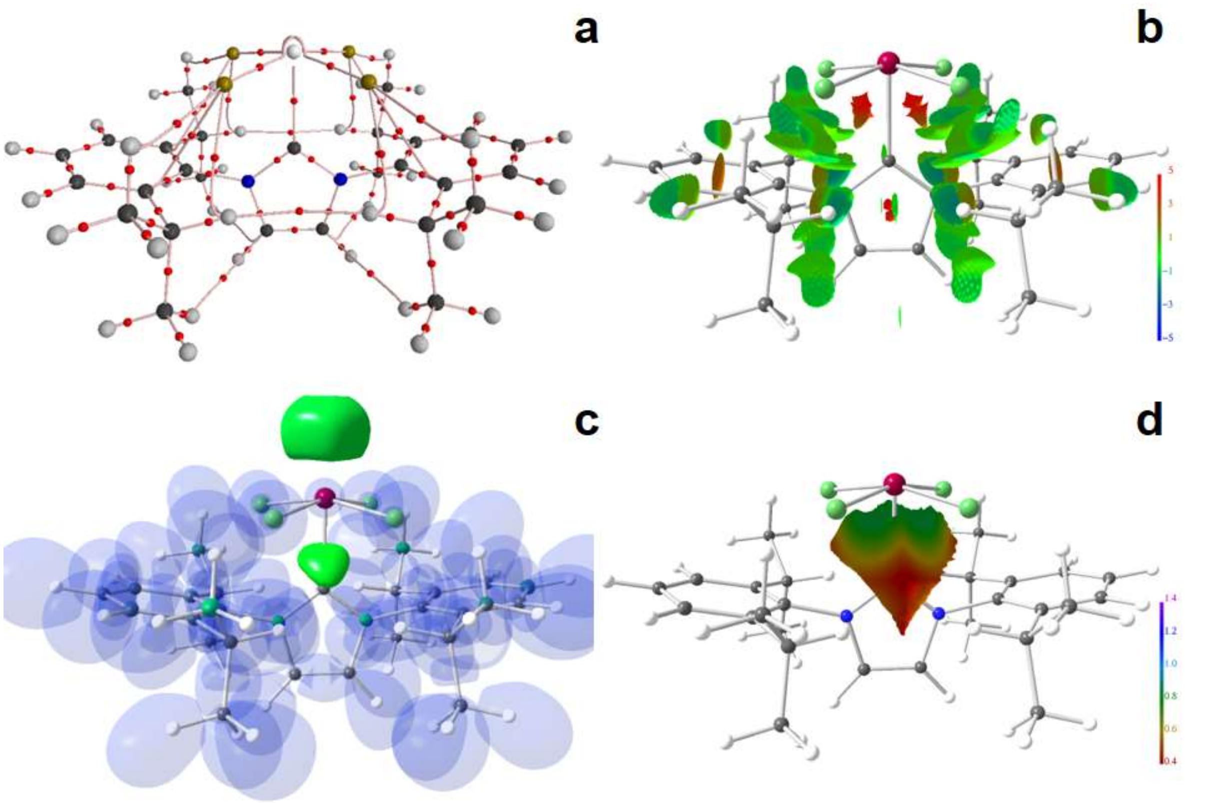

Figure S25 RSBI analysis of experimentally unobserved $IPrTeF_4$ (a) AIM bond paths motif, (b) NCI *iso*-surface at s(**r**) = 0.5, (c) ELI-D localization domain representation at *iso*-value of 1.3, (d) ELI-D distribution mapped on the Te–C ELI-D bonding basin.

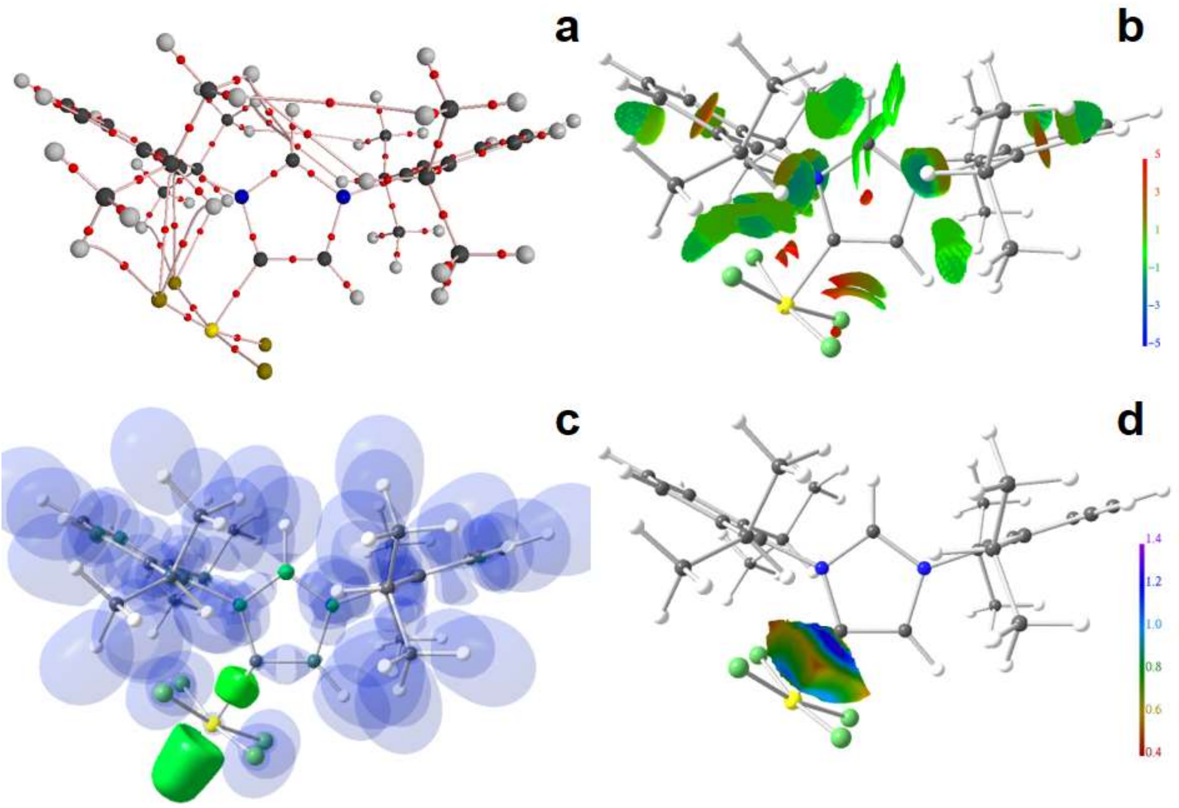

Figure S26 RSBI analysis of experimentally unobserved aIPrSF$_4$ (a) AIM bond paths motif, (b) NCI *iso*-surface at s(**r**) = 0.5, (c) ELI-D localization domain representation at *iso*-value of 1.3, (d) ELI-D distribution mapped on the Se–C ELI-D bonding basin.

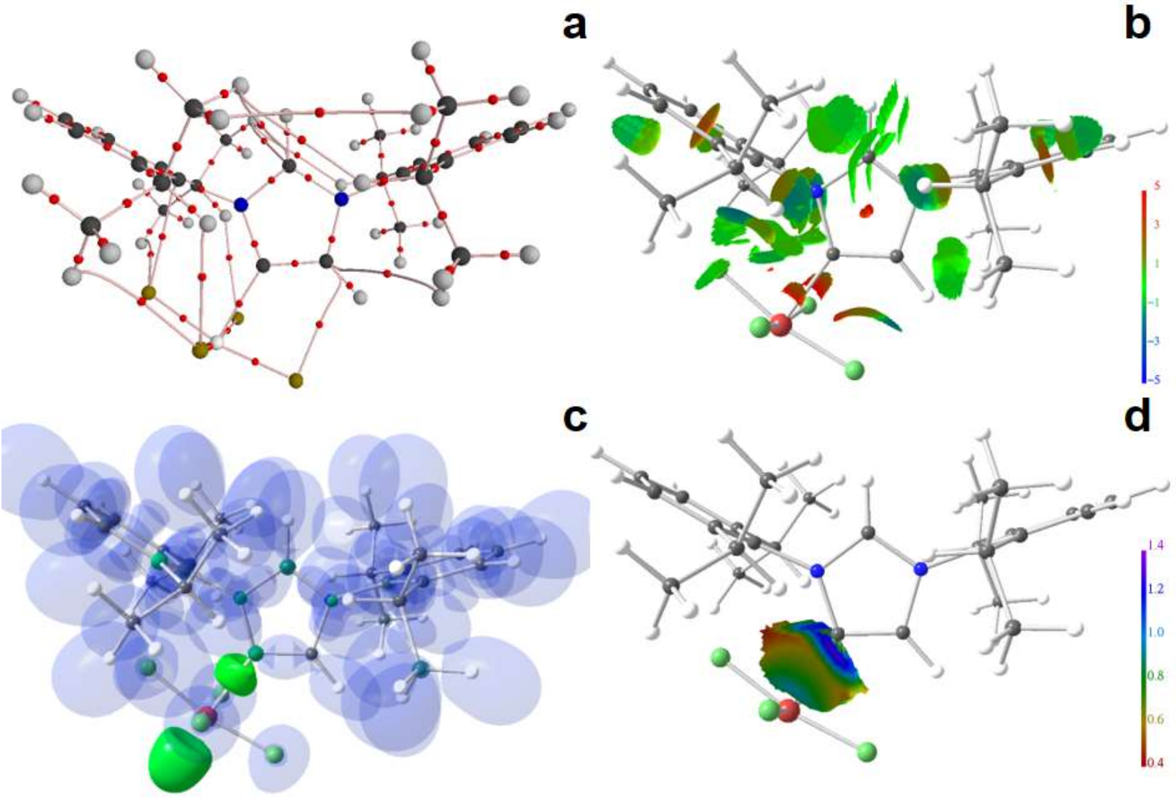

Figure S27 RSBI analysis of experimentally unobserved aIPrSeF_4 (a) AIM bond paths motif, (b) NCI *iso*-surface at s(**r**) = 0.5, (c) ELI-D localization domain representation at *iso*-value of 1.3, (d) ELI-D distribution mapped on the Se–C ELI-D bonding basin.

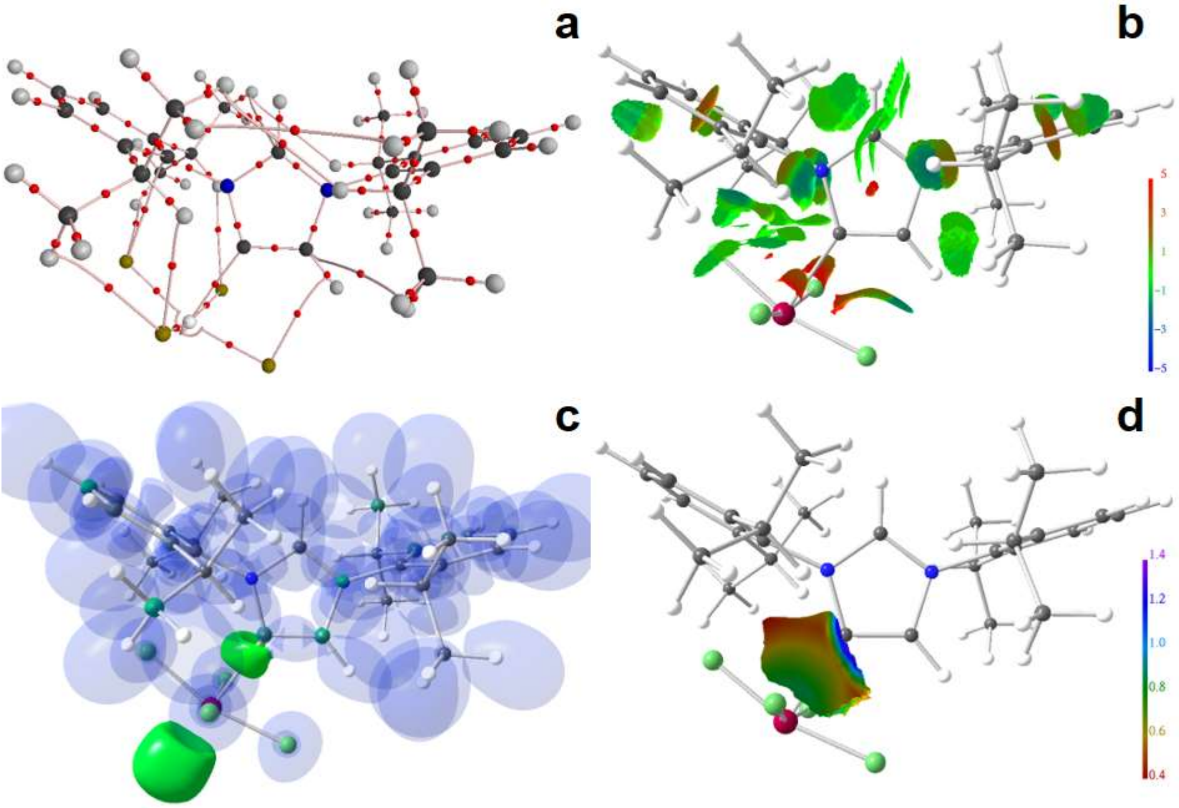

Figure S28 RSBI analysis of **4Te** (a) AIM bond paths motif, (b) NCI *iso*-surface at s(**r**) = 0.5, (c) ELI-D localization domain representation at *iso*-value of 1.3, (d) ELI-D distribution mapped on the Te–C ELI-D bonding basin.

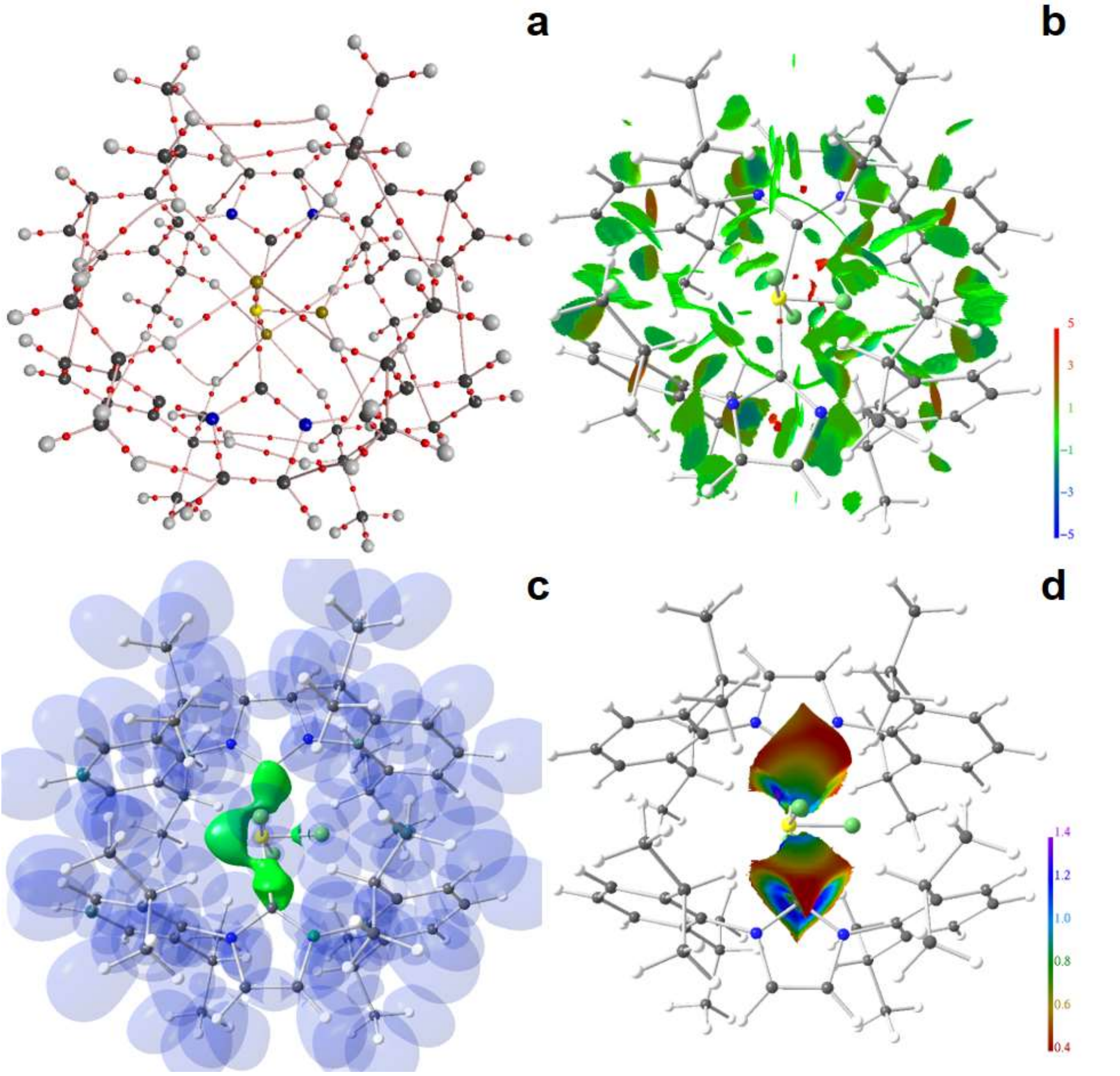

Figure S29 RSBI analysis of experimentally unobserved $[IPr_2SF_3]^+$ (a) AIM bond paths motif, (b) NCI *iso*-surface at s(**r**) = 0.5, (c) ELI-D localization domain representation at *iso*-value of 1.3, (d) ELI-D distribution mapped on the S–C ELI-D bonding basin.

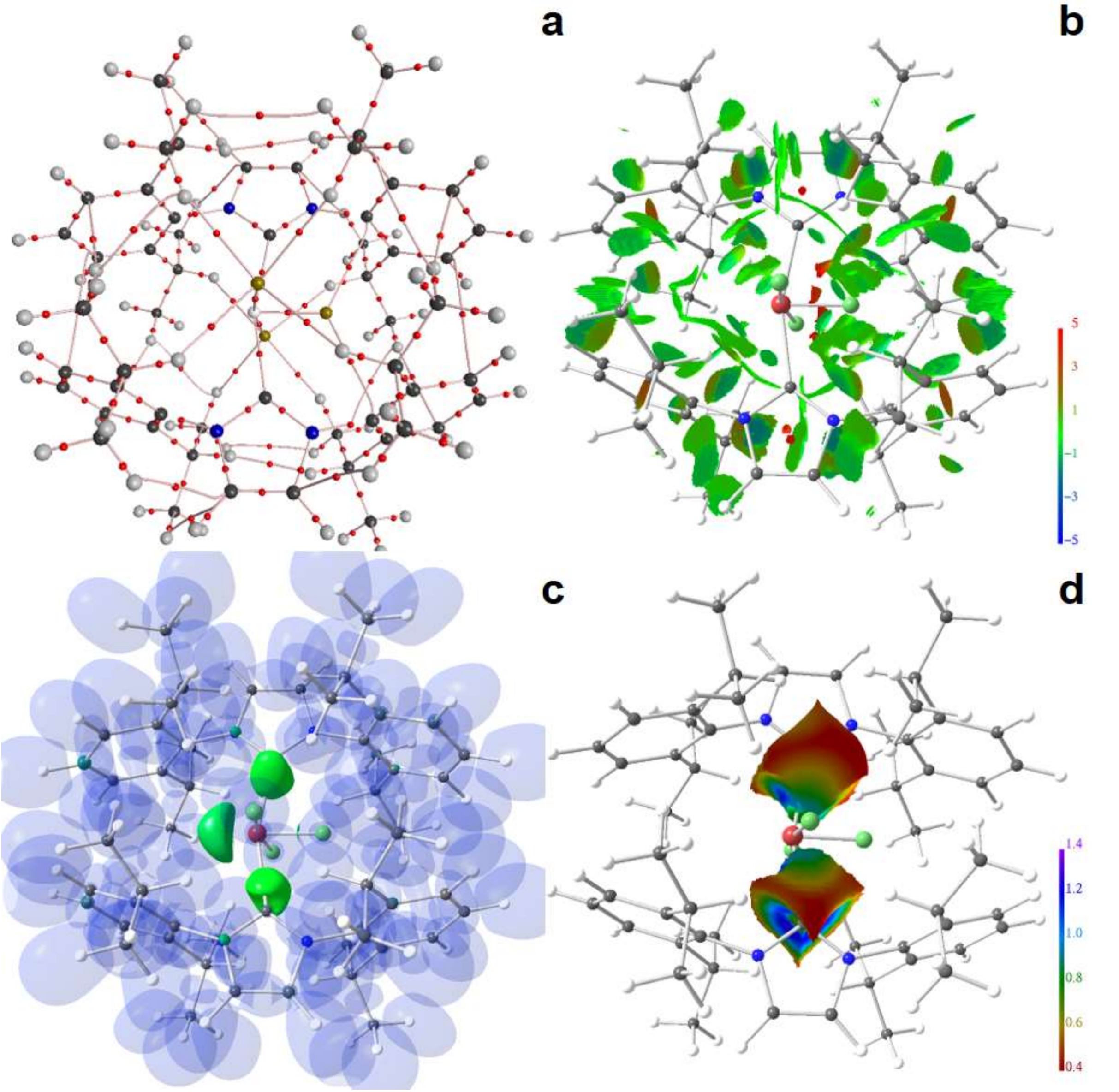

Figure S30 RSBI analysis of $[IPr_2SeF_3]^+$ (a) AIM bond paths motif, (b) NCI *iso*-surface at s(**r**) = 0.5, (c) ELI-D localization domain representation at *iso*-value of 1.3, (d) ELI-D distribution mapped on the Se–C ELI-D bonding basin.

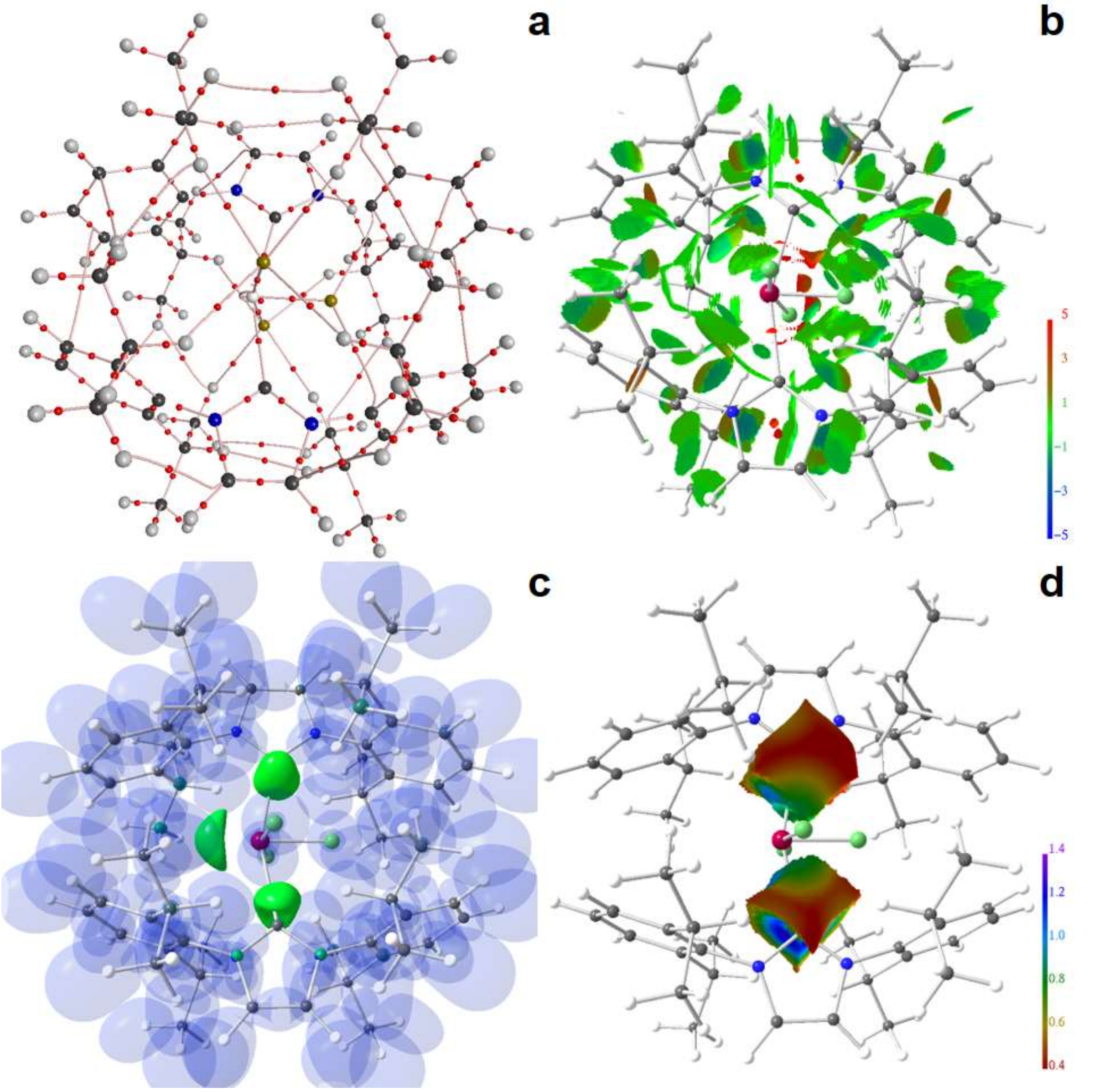

Figure S31 RSBI analysis of **3Te** (a) AIM bond paths motif, (b) NCI *iso*-surface at s(**r**) = 0.5, (c) ELI-D localization domain representation at *iso*-value of 1.3, (d) ELI-D distribution mapped on the Te–C ELI-D bonding basin.

Table S7 Topological and integrated bond properties of the primary E–F bonds

model	contact or basin	d [Å]	$\rho(\mathbf{r})_{bcp}$ [eÅ^{-3}]	$\nabla^2\rho(\mathbf{r})_{bcp}$ [eÅ^{-5}]	ε	$G/\rho(\mathbf{r})_{bcp}$ [a.u.]	$H/(\mathbf{r})_{bcp}$ [a.u.]	N_{ELI} [e]	V_{ELI} [Å^3]	γ_{ELI}
SF_2	S–F	1.604	1.32	2.3	0.51	1.20	–1.08	0.54	0.4	1.37
SF_4	S–Feq	1.563	1.51	–0.6	0.08	1.17	–1.20	0.84	0.7	1.39
$[IPr_2SF_3]^+$	S–F2	1.615	1.36	–2.1	0.08	1.03	–1.13	0.84	0.7	1.42
SF_4	S–Fax	1.665	1.26	–4.2	0.09	0.75	–0.98			
$[IPr_2SF_3]^+$	S–F3	1.732	1.09	–1.5	0.05	0.67	–0.76	0.27	0.3	1.21
$IPrSF_4$	S–F	1.747	1.06	–0.4	0.14	0.66	–0.69	0.26	0.3	1.20
$aIPrSF_4$	S–F3	1.762	1.03	0.0	0.14	0.66	–0.66	0.25	0.3	1.20
$aIPrSF_4$	S–F5	1.769	1.01	0.4	0.15	0.67	–0.64	0.22	0.3	1.19
**2S**	S–F3	1.834	0.83	2.3	0.22	0.71	–0.52	0.06	0.1	1.10
SeF_4	Se–Feq	1.700	1.26	8.4	0.05	1.22	–0.76			
SeF_2	Se–F	1.740	1.11	8.6	0.19	1.22	–0.68			
$[IPr_2SeF_3]^+$	Se–F2	1.742	1.15	7.2	0.03	1.14	–0.71	0.30	0.2	1.41
SeF_4	Se–Fax	1.784	1.06	6.3	0.04	1.07	–0.66			
$[IPr_2SeF_3]^+$	Se–F3	1.843	0.92	5.6	0.03	0.97	–0.55			
**3Se**	Se–F	1.851	0.91	5.5	0.09	0.96	–0.53			
$aIPrSeF_4$	Se–F4	1.872	0.87	5.3	0.10	0.93	–0.50			
**2Se**	Se–F2	1.937	0.72	5.5	0.14	0.93	–0.39			
$aIPrSeF_4$	Se–F2	1.986	0.91	5.6	0.09	0.96	–0.53			
$aIPrSeF_4$	Se–F5	2.001	0.86	5.3	0.10	0.93	–0.50			
$aIPrSeF_4$	Se–F3	2.010	0.86	5.3	0.09	0.92	–0.49			
TeF_4	Te–Feq	1.869	0.97	14.4	0.05	1.41	–0.38			
**3Te**	Te1–F2	1.900	0.91	13.1	0.02	1.37	–0.36			
TeF_2	Te–F	1.914	0.85	12.8	0.11	1.36	–0.31			
TeF_4	Te–Fax	1.933	0.85	11.7	0.03	1.31	–0.35			
$IPrTeF_4$	Te–F	1.983	0.76	9.5	0.07	1.19	–0.31			
**3Te**	Te1–F3	1.985	0.76	9.5	0.02	1.20	–0.31			
**4Te**	Te–F2	1.986	0.75	9.4	0.05	1.19	–0.31			
**4Te**	Te–F4	2.001	0.73	8.9	0.07	1.16	–0.30			
**4Te**	Te–F3	2.010	0.72	8.6	0.07	1.14	–0.30			
$IPrTeF_2$	Te–F3	2.067	0.61	7.5	0.12	1.10	–0.24			

Topological parameters sorted for bond length for each element E. For all bonds, d is the geometric contact distance, $\rho(\mathbf{r})_{bcp}$ is the electron density at the bcp, $\nabla^2\rho(\mathbf{r})_{bcp}$ is the corresponding Laplacian, ε is the bond ellipticity, $G/\rho(\mathbf{r})_{bcp}$ and $H/\rho(\mathbf{r})_{bcp}$ are the kinetic and total energy density over $\rho(\mathbf{r})_{bcp}$ ratios, N_{ELI} and V_{ELI} are electron populations and volumes of related ELI-D basins, γ_{ELI} is the ELI-D value at the attractor position.

Table S8 Topological and integrated bond properties of the primary E–C bonds

model	contact or basin	d [Å]	ρ(**r**)$_{bcp}$ [eÅ^{-3}]	∇^2ρ(**r**)$_{bcp}$ [eÅ^{-5}]	ε	G/ρ(**r**)$_{bcp}$ [a.u.]	H/(**r**)$_{bcp}$ [a.u.]	N_{ELI} [e]	V_{ELI} [Å^3]	γ_{ELI}	RJI [%]
**2S**	S–C	1.727	1.45	−11.0	0.43	0.38	−0.91	2.20	3.7	1.84	73
aIPrSF$_4$	S–C	1.805	1.38	−9.8	0.04	0.21	−0.71	2.30	3.7	2.00	57
IPrSF$_4$	S–C	1.842	1.29	−8.6	0.04	0.21	−0.68	2.45	4.8	2.01	63
[IPr$_2$SF$_3$]$^+$	S–C5	1.995	0.94	−2.6	0.00	0.30	−0.50	2.58	7.1	1.89	73
**2Se**	Se–C	1.878	1.14	−2.9	0.34	0.56	−0.73	2.33	4.8	1.83	76
aIPrSeF$_4$	Se–C	1.941	1.11	−5.4	0.04	0.34	−0.68	2.41	4.7	1.94	62
**3Se**	Se–C	1.986	1.03	−4.4	0.04	0.34	−0.64	2.61	6.3	1.99	67
[IPr$_2$SeF$_3$]$^+$	Se–C5	2.132	0.77	−1.2	0.00	0.36	−0.48	2.62	8.3	1.92	76
IPrTeF$_2$	Te–C	2.099	0.83	2.0	0.31	0.69	−0.53	2.38	6.0	1.88	83
**4Te**	Te–C	2.136	0.84	−0.5	0.04	0.52	−0.55	2.41	6.2	1.95	74
IPrTeF$_4$	Te–C	2.195	0.76	−0.1	0.04	0.50	−0.52	2.58	7.9	2.02	78
**3Te**	Te1–C5	2.295	0.62	0.3	0.01	0.47	−0.44	2.60	9.5	1.97	82
**IPr**	LP(C)							2.44	15.1	2.53	100

See Table S7 for legend

Table S9 ELI-D properties of the S, Se, and Te lone pair basins

	S			Se			Te		
model	N_{ELI} [e]	V_{ELI} [Å^3]	γ_{ELI}	N_{ELI} [e]	V_{ELI} [Å^3]	γ_{ELI}	N_{ELI} [e]	V_{ELI} [Å^3]	γ_{ELI}
**EF$_2$**	2.23	29.0	2.65	2.33	35.0	2.50	2.25	45.5	2.41
**EF$_2$**	2.23	29.0	2.65	2.33	34.9	2.50	2.25	45.4	2.41
IPrEF$_2$	2.26	16.2	2.29	2.31	21.0	2.15	2.23	30.9	2.10
IPrEF$_2$	2.26	16.2	2.29	2.31	21.0	2.15	2.23	30.9	2.10
[IPr$_2$EF$_3$]$^+$	2.09	8.1	2.39	2.24	9.8	2.01	2.16	12.3	1.79
**EF$_4$**	2.31	10.6	2.64	2.43	12.5	2.27	2.25	15.8	2.02
IPrEF$_4$	2.31	8.8	2.62	2.37	10.4	2.36	2.13	13.6	2.25
aIPrEF$_4$	2.40	23.1	2.59	2.44	33.2	2.33	2.19	53.6	2.25

See Table S7for legend

Table S10 Topological and integrated bond properties of the secondary F–Cπ bonds

model	contact	d [Å]	d1 [Å]	d1+d2 [Å]	ρ(**r**)$_{bcp}$ [eÅ^{-3}]	∇^2ρ(**r**)$_{bcp}$ [eÅ^{-5}]	ε	G/ρ(**r**)$_{bcp}$ [a.u.]	H/(**r**)$_{bcp}$ [a.u.]
aIPrSeF$_4$	F2–C42	2.590	1.279	2.592	0.12	1.6	0.25	0.87	0.07
**4Te**	F2–C42	2.700	1.323	2.706	0.10	1.4	0.51	0.85	0.09
[IPr$_2$SF$_3$]$^+$	F2–C12	2.712	1.317	2.714	0.09	1.3	0.41	0.88	0.11
[IPr$_2$SeF$_3$]$^+$	F2–C12	2.766	1.339	2.774	0.09	1.2	0.60	0.86	0.12
IPrSF$_4$	F2–C42	2.815	1.372	2.832	0.08	1.2	1.60	0.88	0.12
**3Te**	F2–C12	2.839	1.366	2.860	0.08	1.1	0.99	0.83	0.12
IPrSeF$_4$	F2–C42	2.881	1.392	2.926	0.08	1.1	1.87	0.85	0.14
aIPrSF$_4$	F2–C42	2.884	1.407	2.905	0.07	1.1	1.70	0.86	0.14
IPrTeF$_4$	F2–C47	2.889	1.412	2.949	0.07	1.0	2.07	0.84	0.14

See Table S7 for legend. d1 and d2 are the distance between atom 1 or 2 and the bcp

Table S11 Topological and integrated bond properties of the secondary F–H bonds

model	contact	d [Å]	d1 [Å]	d1+d2 [Å]	$\rho(\mathbf{r})_{bcp}$ [eÅ^{-3}]	$\nabla^2\rho(\mathbf{r})_{bcp}$ [eÅ^{-5}]	ε	$G/\rho(\mathbf{r})_{bcp}$ [a.u.]	$H/(\mathbf{r})_{bcp}$ [a.u.]
aIPrSeF$_4$	F3–H8	2.223	1.272	2.327	0.13	2.1	2.72	1.01	0.13
**4Te**	F3–H8	2.264	1.304	2.330	0.11	1.7	1.15	0.95	0.13
IPrTeF$_2$	F3–H28	2.331	1.368	2.338	0.08	1.0	0.11	0.82	0.10
**2Se**	F3–H28	2.359	1.377	2.367	0.07	0.9	0.13	0.82	0.10
IPrTeF$_4$	F2–H65	2.382	1.368	2.397	0.07	1.0	0.28	0.86	0.12
**2Se**	F3–H28	2.395	1.388	2.405	0.07	0.9	0.16	0.83	0.11
**3Se**	F2–H65	2.409	1.377	2.426	0.06	0.9	0.35	0.87	0.13
aIPrSF$_4$	F5–H57	2.425	1.380	2.444	0.08	1.1	0.35	0.89	0.13
IPrSF$_4$	F2–H65	2.439	1.384	2.459	0.06	0.9	0.44	0.88	0.14
$[IPr_2SF_3]^+$	F4–H24	2.445	1.441	2.446	0.06	0.7	0.06	0.77	0.10
$[IPr_2SeF_3]^+$	F4–H24	2.446	1.443	2.447	0.06	0.7	0.06	0.78	0.11
**2Se**	F2–H33	2.451	1.453	2.455	0.06	0.8	0.31	0.78	0.10
$[IPr_2SF_3]^+$	F4–H53	2.458	1.445	2.460	0.06	0.7	0.04	0.77	0.10
aIPrSeF$_4$	F5–H57	2.461	1.439	2.464	0.06	0.8	0.12	0.80	0.11
IPrSF$_4$	F2–H64	2.461	1.383	2.490	0.07	1.1	0.57	0.92	0.15
**3Te**	F4–H24	2.464	1.448	2.465	0.06	0.7	0.07	0.78	0.11
**3Te**	F3–H36	2.467	1.457	2.468	0.05	0.7	0.02	0.78	0.12
**3Te**	F3–H64	2.475	1.455	2.478	0.05	0.7	0.02	0.79	0.12
**3Se**	F2–H64	2.477	1.389	2.512	0.07	1.1	0.68	0.92	0.15
$[IPr_2SeF_3]^+$	F4–H53	2.480	1.453	2.483	0.06	0.7	0.04	0.78	0.11
$[IPr_2SeF_3]^+$	F3–H36	2.489	1.460	2.490	0.05	0.6	0.02	0.79	0.13
IPrTeF$_2$	F2–H33	2.489	1.474	2.495	0.06	0.8	0.39	0.78	0.11
**2S**	F2–H33	2.493	1.464	2.496	0.06	0.8	0.32	0.80	0.11
$[IPr_2SeF_3]^+$	F3–H64	2.500	1.456	2.504	0.05	0.7	0.03	0.81	0.13
aIPrSF$_4$	F5–H63	2.501	1.417	2.525	0.05	0.8	0.55	0.88	0.15
**4Te**	F5–H57	2.509	1.459	2.513	0.06	0.7	0.15	0.80	0.11
IPrTeF$_4$	F2–H64	2.513	1.404	2.556	0.07	1.1	0.83	0.93	0.16
$[IPr_2SF_3]^+$	F3–H36	2.526	1.471	2.529	0.05	0.6	0.04	0.80	0.13
aIPrSeF$_4$	F4–H64	2.532	1.489	2.534	0.05	0.6	0.15	0.77	0.12
**3Te**	F4–H53	2.535	1.475	2.541	0.05	0.7	0.04	0.79	0.13
**4Te**	F4–H64	2.614	1.527	2.616	0.04	0.6	0.20	0.78	0.13
**2S**	F3–H26	2.668	1.497	2.695	0.05	0.7	0.46	0.90	0.18
**2Se**	F3–H26	2.686	1.507	2.717	0.05	0.7	0.58	0.90	0.19
IPrTeF$_2$	F2–H63	2.729	1.368	2.339	0.08	1.0	0.11	0.82	0.10
IPrTeF$_2$	F3–H26	2.730	1.527	2.767	0.04	0.7	0.89	0.91	0.21
**4Te**	F5–H63	2.734	1.539	2.754	0.04	0.5	0.50	0.87	0.19
**4Te**	F2–H68	2.753	1.572	2.767	0.03	0.5	0.43	0.85	0.19
aIPrSeF4	F5–H63	2.818	1.575	2.858	0.03	0.5	1.54	0.90	0.23
aIPrSeF4	F2–H68	2.886	1.632	2.936	0.02	0.4	4.37	0.90	0.26

See Table S7 for legend. d1 and d2 are the distance between atom 1 or 2 and the bcp

Table S12 AIM atomic and fragmental charges (in e)

model	E	F(1)	F(2)	F(3)	F(4)	NHC(1)	NHC(2)
**2S**	0.84	–0.68	–0.68			0.52	
**2Se**	0.93	–0.71	–0.71			0.49	
$IPrTeF_2$	1.17	–0.77	–0.77			0.37	
**SF2**	1.24	–0.62	–0.62				
**SeF2**	1.16	–0.58	–0.58				
**TeF2**	1.30	–0.65	–0.65				
$[IPr_2SF_3]^+$	1.79	–0.67	–0.66	–0.66		0.10	0.10
$[IPr_2SeF_3]^+$	1.94	–0.64	–0.68	–0.68		0.03	0.03
**3Te**	2.30	–0.70	–0.73	–0.73		–0.07	–0.07
$aIPrSF_4$	1.83	–0.64	–0.64	–0.66	–0.66	0.77	
$aIPrSeF_4$	2.05	–0.68	–0.68	–0.68	–0.68	0.67	
**4Te**	2.44	–0.73	–0.73	–0.74	–0.74	0.50	
$IPrSF_4$	1.87	–0.65	–0.65	–0.66	–0.66	0.75	
**4Te**	2.10	–0.68	–0.68	–0.68	–0.68	0.62	
IPrTeF4	2.47	–0.74	–0.74	–0.74	–0.74	0.49	
**SF4**	2.52	–0.63	–0.63	–0.63	–0.63		
**SeF4**	2.40	–0.57	–0.57	–0.63	–0.63		
**TeF4**	2.68	–0.63	–0.63	–0.71	–0.71		

References

S1 L. Hintermann, *Beilstein J. Org. Chem.* **2007**, *3*, DOI: 10.1186/1860-5397-3-22.

S2 M. Pompeo, R. D. J. Froese, N. Hadei, M. G. Organ, *Angew. Chem. Int. Ed.* **2012**, *51*, 11354–11357.

S3 S. Wei, X.-G. Wei, X. Su, J. You, Y. Ren, *Chem. Eur. J.,* **2011**, *17*, 5965–5971.

S4 D. J. Nelson, A. Collado, S. Manzini, S. Meiries, A. M. Z. Slawin, D. B. Cordes, S. P. Nolan, *Organometallics* **2014**, *33*, 2048–2058.

S5 D. Rottschäfer, D. E. Fuhs, B. Neumann, H.-G.; Stammler, R. S. Ghadwal, *Z. Anorg. Allg. Chem.* **2020**, *646*, 574–579.

S6 G. Talavera, J. Peña, M. Alcarazo, *J. Am. Chem. Soc.* **2015**, *137*, 8704–8707.

S7 G. R. Fulmer, A. J. M. Miller, N. H. Sherden, H. E. Gottlieb, A. Nudelman, B. M. Stoltz, J. E. Bercaw, K. I. Goldberg, *Organometallics* **2010**, *29*, 2176–2179.

S8 N. W. Goldberg, X. Shen, J. Li, T. Ritter, *Org. Lett.* **2016**, *18*, 6102–6104.

S9 (a) K. O. Christe, D. A. Dixon, R. Haiges, M. Hopfinger, V. E. Jackson, T. M. Klapötke, B. Krumm, M. Scherr, *Journal of Fluorine Chemistry* **2010**, *131*, 791–799. (b) A. R. Mahjoub, D. Leopold, K. Seppelt, *Z. Anorg. Allg. Chem.* **1992**, *618*, 83–88.

S10 N. Kuhn, H. Bohnen, J. Fahl, D. Bläser, R. Boese, *Chem. Ber.* **1996**, *129*, 1579–1586.

S11 G. M. Sheldrick, *Acta Cryst.*, **2008**, *A64*, 112.

S12 L. Farrugia, *J. Appl. Cryst.*, **1999**, *32*, 837.

S13 O. V. Dolomanov, L. J. Bourhis, R. J. Gildea, J. A. K. Howard, H. Puschmann, *J. Appl. Cryst.* **2009**, *42*, 339–341.

S14 K. Brandenburg, Diamond, version 4.0.4, Crystal Impact GbR: Bonn, Germany, **2012**.

S15 (a) A. D. Becke, *J. Chem. Phys.*, **1993**, *98*, 5648–5652. (b) J. P. Perdew, J. A. Chevary, S. H. Vosko, K. A. Jackson, M. R. Pederson, D. J. Singh, C. Fiolhais, *Phys. Rev. B*, **1992**, *46*, 6671–6687.

S16 M. J. Frisch, G. W. Trucks, H. B. Schlegel, G. E. Scuseria, M. A. Robb, J. R. Cheeseman, G. Scalmani, V. Barone, G. A. Petersson, H. Nakatsuji, X. Li, M. Caricato, A. V. Marenich, J. Bloino, B. G. Janesko, R. Gomperts, B. Mennucci, H. P. Hratchian, J. V. Ortiz, A. F. Izmaylov, J. L. Sonnenberg, D. Williams-Young, F. Ding, F. Lipparini, F. Egidi, J. Goings, B. Peng, A. Petrone, T. Henderson, D. Ranasinghe, V. G. Zakrzewski, J. Gao, N. Rega, G.

Zheng, W. Liang, M. Hada, M. Ehara, K. Toyota, R. Fukuda, J. Hasegawa, M. Ishida, T. Nakajima, Y. Honda, O. Kitao, H. Nakai, T. Vreven, K. Throssell, J. A. Montgomery, Jr., J. E. Peralta, F. Ogliaro, M. J. Bearpark, J. J. Heyd, E. N. Brothers, K. N. Kudin, V. N. Staroverov, T. A. Keith, R. Kobayashi, J. Normand, K. Raghavachari, A. P. Rendell, J. C. Burant, S. S. Iyengar, J. Tomasi, M. Cossi, J. M. Millam, M. Klene, C. Adamo, R. Cammi, J. W. Ochterski, R. L. Martin, K. Morokuma, O. Farkas, J. B. Foresman, and D. J. Fox, Gaussian, Inc., Wallingford CT, **2016**.

[S17] a) K. A. Peterson, *J. Chem. Phys.* **2003**, *119*, 11099–11112. b) K. A. Peterson, D.Figgen, E. Goll, H. Stoll, M. Dolg, *J. Chem. Phys*. **2003**, *119*, 11113–11123.

[S18] S.; Grimme, J. Anthony, S. Ehrlich, H. Krieg, *J. Chem. Phys.* **2010**, *132*, 154104–154123.

[S19] F. M.; Bickelhaupt, E. J. Baerends, *Rev. Comput. Chem.* **2000**, *15*, 1–86.

[S20] (a) G. te Velde, F. M. Bickelhaupt, E. J. Baerends, C. Fonseca Guerra, S. J. A. van Gisbergen, J. G. Snijders, T. Ziegler, *J. Comput. Chem.* **2001**, *22*, 931–967. (b) E. J. Baerends, T. Ziegler, A. J. Atkins, J. Autschbach, O. Baseggio, D. Bashford, A. Bérces, F. M. Bickelhaupt, C. Bo, P. M. Boerrigter, C. Cappelli, L. Cavallo, C. Daul, D. P. Chong, D. V. Chulhai, L. Deng, R. M. Dickson, J. M. Dieterich, F. Egidi, D. E. Ellis, M. van Faassen, L. Fan, T. H. Fischer, A. Förster, C. Fonseca Guerra, M.; Franchini, A. Ghysels, A. Giammona, S. J. A. van Gisbergen, A. Goez, A. W. Götz, J. A. Groeneveld, O. V. Gritsenko, M. Grüning, S. Gusarov, F. E. Harris, P. van den Hoek, Z. Hu, C. R. Jacob, H. Jacobsen, L. Jensen, L. Joubert, J. W. Kaminski, G. van Kessel, C. König, F. Kootstra, A. Kovalenko, M. V. Krykunov, P. Lafiosca, E. van Lenthe, D. A. McCormack, M. Medves, A. Michalak, M. Mitoraj, S. M. Morton, J. Neugebauer, V. P. Nicu, L. Noodleman, V. P. Osinga, S. Patchkovskii, M. Pavanello, C. A. Peeples, P. H. T. Philipsen, D. Post, C. C. Pye, H. Ramanantoanina, P. Ramos, W. Ravenek, M. Reimann, J. I. Rodríguez, P. Ros, R. Rüger, P. R. T. Schipper, D. Schlüns, H. van Schoot, G. Schreckenbach, J. S. Seldenthuis, M. Seth, J. G. Snijders, M. Solà, M. Stener, M. Swart, D. Swerhone, V. Tognetti, G. te Velde, P. Vernooijs, L. Versluis, L. Visscher, O. Visser, F. Wang, T. A. Wesolowski, E. M. van Wezenbeek, G. Wiesenekker, S. K. Wolff, T. K. Woo, A. L. Yakovlev, ADF 2021.1, SCM,

Theoretical Chemistry, Vrije Universiteit, Amsterdam, The Netherlands, http://www.scm.com. (accessed August 17, 2021)

[S21] (a) C. T. Campos, F. E. Jorge, *Mol. Phys.* **2013**, *111*, 167−173. (b) E. van Lenthe, E. J. Baerends, *J. Comput. Chem.* **2003**, *24*, 1142−1156.

[S22] R. W. F. Bader, *Atoms in Molecules: A Quantum Theory* (Cambridge University Press: Oxford U.K., 1991).

[S23] F. Biegler-König, J. Schönbohm, D. Bayles, *J. Comput. Chem*., **2001**, *22*, 545–559.

[S24] M. Kohout, DGRID-4.6 Radebeul, **2015**.

[S25] M. Kohout, *Int. J. Quantum Chem.*, **2004**, *97*, 651–658.

[S26] M. Kohout, F. R. Wagner, Y. Grin, *Theor. Chem. Acc.*, **2008**, *119*, 413–420.

[S27] E. R. Johnson, S. Keinan, P. Mori-Sanchez, J. Contreras-García, A. J. Cohen, W. Yang, *J. Am. Chem. Soc.*, **2010**, *132*, 6498–6506.

[S28] J. Contreras-García, E. Johnson, S. Keinan, R. Chaudret, J.-P. Piquemal, D. Beratan, W. Yang, *J. Chem. Theor. Comp.*, **2011**, *7*, 625–632.

[S29] C. B. Hübschle, P. Luger, *J. Appl. Crystallogr*.**,** **2006**, *39*, 901–904.
